# Conjunctival melanoma copy number alterations and correlation with mutation status, tumor features, and clinical outcome

**DOI:** 10.1111/pcmr.12767

**Published:** 2019-02-19

**Authors:** Nihal Kenawy, Helen Kalirai, Joseph J. Sacco, Sarah L. Lake, Steffen Heegaard, Ann‐Cathrine Larsen, Paul T. Finger, Tatyana Milman, Kimberly Chin, Carlo Mosci, Francesco Lanza, Alexandre Moulin, Caroline A. Schmitt, Jean Pierre Caujolle, Célia Maschi, Marina Marinkovic, Azzam F. Taktak, Heinrich Heimann, Bertil E. Damato, Sarah E. Coupland

**Affiliations:** ^1^ Liverpool Ocular Oncology Research Group Institute of Translational Medicine University of Liverpool Liverpool UK; ^2^ Aintree University Hospital Liverpool UK; ^3^ Clatterbridge Cancer Centre Wirral UK; ^4^ Eye Pathology Section, Department of Pathology and Department of Ophthalmology Rigshospitalet, University of Copenhagen Copenhagen Denmark; ^5^ The New York Eye Cancer Centre New York; ^6^ Ocular Oncology Service Galliera Hospital Genoa Italy; ^7^ Ophthalmic Pathology Laboratory and Department of Ophthalmology Jules Gonin Eye Hospital Lausanne Switzerland; ^8^ Ophthalmology Department Oslo University Hospital Oslo Norway; ^9^ Ophthalmology Department University Hospital of Nice Nice France; ^10^ Ophthalmology Department Leiden University Medical Centre Leiden The Netherlands; ^11^ Department of Medical Physics and Clinical Engineering Royal Liverpool University Hospital Liverpool UK; ^12^ Liverpool Ocular Oncology Centre Royal Liverpool University Hospital Liverpool UK; ^13^ Oxford Eye Hospital and Nuffield Department of Clinical Neurosciences University of Oxford Oxford UK; ^14^ Cellular Pathology Liverpool Clinical Laboratories Royal Liverpool University Hospital Liverpool UK

**Keywords:** allele‐specific copy number, *BRAF/NRAS* mutation, conjunctival melanoma, copy number alteration, metastasis

## Abstract

Relatively little is known about the genetic aberrations of conjunctival melanomas (CoM) and their correlation with clinical and histomorphological features as well as prognosis. The aim of this large collaborative multicenter study was to determine potential key biomarkers for metastatic risk and any druggable targets for high metastatic risk CoM. Using Affymetrix single nucleotide polymorphism genotyping arrays on 59 CoM, we detected frequent amplifications on chromosome (chr) 6p and deletions on 7q, and characterized mutation‐specific copy number alterations. Deletions on chr 10q11.21‐26.2, a region harboring the tumor suppressor genes, *PDCD4*,* SUFU*,* NEURL1*,* PTEN*, *RASSF4*,* DMBT1*, and* C10orf90* and* C10orf99*, significantly correlated with metastasis (Fisher's exact, *p* ≤ 0.04), lymphatic invasion (Fisher's exact, *p* ≤ 0.02), increasing tumor thickness (Mann–Whitney, *p* ≤ 0.02), and *BRAF* mutation (Fisher's exact, *p* ≤ 0.05). This enhanced insight into CoM biology is a step toward identifying patients at risk of metastasis and potential therapeutic targets for systemic disease.

**Significance**

Conjunctival melanoma (CoM) is a rare but potentially fatal melanoma subtype. We analyzed copy number alterations, and their frequencies, in relation to tumor characteristics and patients’ outcomes. We identified recurrent 10q deletions, which correlated with histological features of poor prognosis and metastatic risk. This finding should facilitate future development of disease‐specific prognostic and therapeutic models.

## 1 INTRODUCTION

Conjunctival melanoma (CoM) is a rare but potentially fatal ocular cancer (Kenawy, Lake, Coupland, & Damato, 2013). Local CoM recurrence occurs in 5%–26% of cases after local excision and brachytherapy with/without cryotherapy (Damato & Coupland, 2009; Missotten, Keijser, De Keizer, & De Wolff‐Rouendaal, 2005; Shields et al., 2000; Werschnik & Lommatzsch, 2002); however, recurrence occurs in over 50% when treated with surgical excision alone (Shields et al., 2000; Tuomaala, Eskelin, Tarkkanen, & Kivelä, 2002). Regional lymph node metastasis occurs in 15%–41% by a median of 2.3 years post‐diagnosis, whereas systemic metastases (± regional nodes involvement) develop in 9%–25%, by just over 3 years. The 10‐year CoM‐related mortality is 18%–30% (Damato & Coupland, 2008; Shields et al., 2000; Tuomaala & Kivela, 2004; Werschnik & Lommatzsch, 2002). Clinical and pathological predictors of metastasis include the following: non‐bulbar tumor location, local tumor recurrence, epithelioid cell morphology, and a high mitotic count (Seregard, 1993; Shields et al., 2000; Tuomaala et al., 2002). The molecular drivers of metastasis are largely unknown in CoM because of its rarity and because of the usual paucity of tissue available for detailed analysis.

Previous studies investigating CoM genetic abnormalities had variable, and often small, cohort sizes (*n* = 16–78), and mostly targeted hotspot mutations known to occur in cutaneous melanoma (CM), namely in *BRAF*, *NRAS*, *KIT,* and the *TERT *promoter described in on average 40%–50%, 15%–25%, 1%–3%, and ~70% of CM cases, respectively (Broekaert et al., 2010; Carr & Mackie, 1994; Curtin, Busam, Pinkel, & Bastian, 2006; Davies et al., 2002; Handolias et al., 2010; Horn et al., 2013; Huang et al., 2013; Moltara et al., 2018; van 't Veer et al., 1989). In CoM, such mutations are reported in 8%–54%, 0%–18%, 0%–11%, and 32%, respectively (Beadling et al., 2008; El‐Shabrawi, Radner, Muellner, Langmann, & Hoefler, 1999; Gear, Williams, Kemp, & Roberts, 2004; Goldenberg‐Cohen et al., 2005; Griewank et al., 2013; Lake et al., 2011; Larsen et al., 2015; Populo, Soares, Rocha, Silva, & Lopes, 2010; Scholz et al., 2018; Spendlove et al., 2004). Whole exome sequencing on a relatively small series of 5 CoMs identified mutually exclusive *NF1*,* BRAF*, and *NRAS *driver mutations in 20%, 60%, and 20% of samples, respectively, alongside other individual cancer‐associated and epigenetic regulator mutations, such as those of *EGFR* and the *TERT* promoter (Swaminathan et al., 2017). Most recently, a larger study using next‐generation sequencing discovered mutations of *NF1* in 21 of 63 (33%) CoMs, *BRAF* in 16 (25%), *NRAS* in 11 (17%), and *KRAS* in a single sample (Scholz et al., 2018). Mutations in *NF1* were mostly mutually exclusive with those in *BRAF* or *NRAS* although exact frequencies were not given (Scholz et al., 2018). These recent findings are also consistent with CM where *NF1* mutations occur in 12%–30%, and are generally mutually exclusive from tumors with *BRAF* and *NRAS *mutations (Cirenajwis et al., 2017; Hodis et al., 2012; Krauthammer et al., 2015). In line with CM, a 4‐group gene‐specific mutation/triple wild type (wt) classification for CoM was proposed (Cancer Genome Atlas, Network, 2015; Scholz et al., 2018).

In addition to mutations, gross or regional chromosomal copy number alterations (CNAs) in CoM have been reported including gains of 1q, 3, 4q, 6p, 8,11, 12p, 13q, 14p, 17q, and 22q and losses of 1p, 3q, 8p, 9p, 10, 11q, 12q, 13, 15p, 16p, and 17p (Dahlenfors, Tornqvist, Wettrell, & Mark, 1993; Griewank et al., 2013; McNamara, Felix, Davison, Fenton, & Kennedy, 1997; Swaminathan et al., 2017; Vajdic et al., 2003). We have previously demonstrated chromosome (chr) 6p regional amplification using multiplex ligation‐dependent probe amplification (MLPA), which detected *CDKN1A* and *RUNX2 *(both on 6p21.2) gains in 69% of 16 and 76% of 21 primary CoMs, respectively, and 75% and 100% of 4 metastatic CoM, respectively (Lake et al., 2011). We also identified amplifications of *MLH1 *(3p22.1) and *TIMP2* (17q25.3) in 75% of 4 and 83% of 6 metastatic CoM, respectively, as well as *MGMT* and* ECHS1* (both on 10q26.3) deletions in 83% of the six metastatic samples (Lake et al., 2011).

However, the overall prevalence of these CNAs in CoM and their correlation with disease characteristics and prognosis remain unclear. Lake et al did not reveal any association between 6p21.2 gains and histological cell type, age, sex, or survival (Lake et al., 2011). In addition, no correlation between *BRAF, NRAS* or *NF1* mutations and recurrence, metastasis, or mortality was found (Gear et al., 2004; Griewank et al., 2013; Lake et al., 2011; Larsen et al., 2016; Scholz et al., 2018; Sheng et al., 2015). A strong association between *BRAF* mutation and sun exposure was determined in two reports (*p* ≤ 0.03; Griewank et al., 2013; Larsen et al., 2016), and with clinical and pathological T1 stage (*p* = 0.007) in a single study (Larsen et al., 2016). The data are divided with regard to *BRAF* mutations in relation to age and sex, with some authors reporting a significant correlation with male gender and age younger than 65 years (*p* ≤ 0.02; Larsen et al., 2016), whereas others did not find any correlation between these parameters (Griewank et al., 2013; Scholz et al., 2018; Sheng et al., 2015).

Hence, there was a clear need to study, in depth, the prevalence of various CNAs and their clinical significance in a large clinically well‐defined CoM cohort with a genome‐wide approach. This current multicenter collaborative project was established to examine one of the largest CoM cohorts using single nucleotide polymorphism (SNP) genotyping array, with the intention of defining key biomarkers of CoM metastatic risk, and to correlate these with clinico‐histological features and clinical outcomes, in order to identify patients at risk of metastasis, and also potential therapeutic targets for systemic disease.

## 2 MATERIALS AND METHODS

### 2.1 Study design

Eight clinical centers were involved in the study: the Liverpool Ocular Oncology Centre (LOOC; the principal site), Liverpool, UK; University of Copenhagen, Denmark; The New York Eye Centre, USA; National Institute of Cancer Research, Genoa, Italy; University of Lausanne, Switzerland; Oslo University Hospital, Norway; St Roche Hospital, Nice, France; and Leiden University, the Netherlands. Ethical approval was obtained from the principal's site Research Ethics Committee (REC reference: 11/NW/0340) and the local health authority of each collaborating center. All patients gave informed consent to participate. The study followed the tenets of the Declaration of Helsinki.

### 2.2 Data collection

Standardized clinical and pathological proformas were used across the centers for data collection at time of recruitment and study closure in March 2016.

Data collected: (a) Patient demographics: age at presentation, sex, ethnicity, duration of follow‐up (FU), time from presentation to metastasis and organ of metastasis, survival status at study closure, and cause of death if deceased. Overall survival (OS) was calculated from date of presentation to end of study or death date; (b) clinical CoM criteria: primary or recurrent, laterality, location and extent in clock hours, number of tumors if more than one, presence of associated lymph node, and/or distal metastases; and (c) histopathological CoM criteria: measurements in mm, presence of epithelioid cells, mitotic count/5 high power fields (HPF), lymphovascular invasion, extension to lateral or deep surgical margins, and if associated with pre‐invasive disease (i.e., either termed “primary acquired melanosis” (PAM) or conjunctival melanocytic intraepithelial neoplasia (C‐MIN; Damato & Coupland, 2008)). For every recruited patient, an FFPE block with a representative H&E slide, color photographs, and a high‐frequency B‐scan ultrasound measurement, if possible, of the CoM(s) were collected.

### 2.3 DNA extraction

For each sample, a 4‐μm formalin‐fixed paraffin‐embedded (FFPE) H&E‐stained section was examined to identify areas with greater than 90% tumor cells. DNA was extracted using QIAamp^®^ DNA Mini Kit (Qiagen, Crawley, UK) following a modified protocol as previously described by Lake et al. (2011). The quality of extracted DNA was determined by a modified multiplex PCR, adapted from van Dongen et al. (2003). PCR products were visualized on 2% agarose gels stained with 1X SYBR Safe (Invitrogen, Paisley, UK) using the BioDoc‐It Imaging System (Ultra‐Violet Products Ltd., Cambridge, UK).

### 2.4 SNP array

Affymetrix SNP 6.0 genotyping (Affymetrix, Santa Clara, CA) was performed at Atlas Biolabs (Berlin, Germany). Raw data were analyzed by Partek Genomics Suite^®^ (PGS) version 6.6 (Partek Incorporated, St. Louis, MO) as previously described by McCarthy et al. (2016).

The SNP data for all tumors can be accessed in the international public repository Gene Expression Omnibus (GEO, http://www.ncbi.nlm.nih.gov/geo; accession number GSE123011).

### 2.5 Mutation detection

*BRAF* mutation was investigated in 53 CoM samples of which 35 were tested in the University of Liverpool Laboratories for *BRAF* V600E/Ec, K, D, and R mutations, using the Qiagen™ Therascreen *BRAF* RGQ PCR Kit (catalogue number 870211), according to the manufacturer's instructions, on a Rotor‐Gene Q real‐time PCR cycler (5plex HRM series). The remaining 18 samples were investigated in the University of Copenhagen by droplet digital PCR (ddPCR), which tests for V600E and K only.

*NRAS* mutation was investigated in 45 of the 53 *BRAF*‐tested samples of which 34 were analyzed with pyrosequencing in triplicate reactions at The Manchester Centre for Genomic Medicine, UK, for any of the known mutations at codons 12, 13, and 61. The remaining 11 samples were analyzed in the University of Copenhagen by denaturing gradient gel electrophoresis (DGGE) for p.Q61K, p.Q61L, and p.Q61R mutations.

### 2.6 Data analysis

Data analysis was undertaken in three separate work packages as outlined below:

1. Global analysis of all CNAs detected. CNAs in the primary and locally recurrent CoMs were initially compared. Gains or losses occurring in at least 40% of the 59 samples were then examined. This was an arbitrary cutoff to reliably identify the most common changes in CoM from the vast spectrum of CNAs obtained by PGS and was chosen after several analyses with higher and lower cutoff levels.
2. Comparison of CNAs in CoM that metastasized (CoMMET+) with those without metastases (CoMMET−) after exclusion of patients with FU less than 3.4 years (the median time to metastasis reported in the literature; Tuomaala & Kivela, 2004). CNAs unique to either group or those shared between at least 50% of the cases in the two groups were identified using Venn diagrams (Oliveros, 2007–2015, http://bioinfogp.cnb.csic.es/tools/venny/index.html). This cutoff allowed the identification of alterations significantly associated with metastatic risk. The lists were further refined to include only known oncogenes and tumor suppressor genes (TSGs) as defined in UniProt (http://www.Uniprot.org).
3. CoMs were categorized according to their *BRAF/NRAS* mutation status as follows: (a) *BRAF*‐mutant (mt), (b) *NRAS*‐mt, and (c) tumors harboring neither mutation, that is, “*BRAF* and *NRAS *wild type (wt).” One sample harbored both *BRAF* and *NRAS* mutations, and was excluded from the analyses. CNAs detected by PGS in the mutant tumors were compared to identify those unique to either mutation as described above. The list was further refined to include only oncogenes and TSGs as defined in UniProt. Comparisons to CoMs that were wild type for both *BRAF *and *NRAS* were not performed as they may have included *NF1* mutations, which we did not test for and would have likely confounded the results. Finally, to assess whether gene dosage was relevant to the mutation status, the amplification frequency of the *BRAF* and *NRAS* genes in the mutant groups was compared.

### 2.7 Immunohistochemistry

The four significantly deleted TSGs, *NEURL1*,* SUFU*, *PDCD4*, and *C10orf90*, identified in CoMs that metastasized were selected for immunohistochemical analyses. Four‐µm thick FFPE tissue sections were used from available CoM samples. Fifteen sections were tested for *NEURL1*, 14 for *SUFU* and *PDCD4*, and 13 for *C10orf90*. Antigen retrieval and staining were performed as previously described by Lake et al. (2013). Primary antibodies were as follows: anti‐NEURL1 (HPA044204, rabbit polyclonal) 1:10 and pancreas positive control; anti‐SUFU (HPA008700, rabbit polyclonal) 1:100 and colon positive control; anti‐PDCD4 (ab105998, mouse monoclonal) 1:400 and testis positive control; and anti‐C10orf90 (HPA038648, rabbit polyclonal) 1:250 and testis positive control. Anti‐NEURL1, SUFU, and C10orf90 were supplied by Atlas Antibodies (Stockholm, Sweden), and anti‐PDCD4 was supplied by Abcam (Cambridge, UK). Tissue sections were scored for localization (nuclear/cytoplasmic/membrane) and intensity of the stain by three investigators (NK, HK, SEC) as previously described by Remmele & Stenger according to the following: (A) percentage of stained tumor cells: 0% (equates to 0), 1%–24% (1), 25%–49% (2), 50%–74% (3), and 75%–100% (4), and (B) intensity of staining: none (0), weak (1), moderate (2), and strong (3) (Remmele & Stegner, 1987). Both A and B were scored for cytoplasm or plasma membrane (PM) staining, and multiplying A and B produced the final score, ranging therefore between 0 and 12. For proteins localized to the nucleus on IHC, without much variability of intensity, only (A), the percentage of positive tumor cells, was evaluated.

### 2.8 Statistical analyses

A Mann–Whitney test was used for non‐parametric continuous variables and Pearson's chi‐square/Fisher's exact for categorical variables. Kaplan–Meier survival curves were used to assess metastasis‐free survival and OS. Independent *t* test was used for immunohistochemistry (IHC) scoring comparisons. All analyses were performed using IBM SPSS Statistics software version 22, IBM, Chicago, IL.

## 3 RESULTS

### 3.1 Patients and demographics

A total of 98 adult patients with invasive CoM were recruited from eight collaborating centers. Only 59 of the 98 FFPE tissue samples yielded sufficient DNA for SNP 6.0 microarray genotyping. The demographics of the 59 patients and their tumor characteristics are summarized in Supporting Information Table S1.

### 3.2 Global analysis of CNAs in all study samples

**Figure 1:**
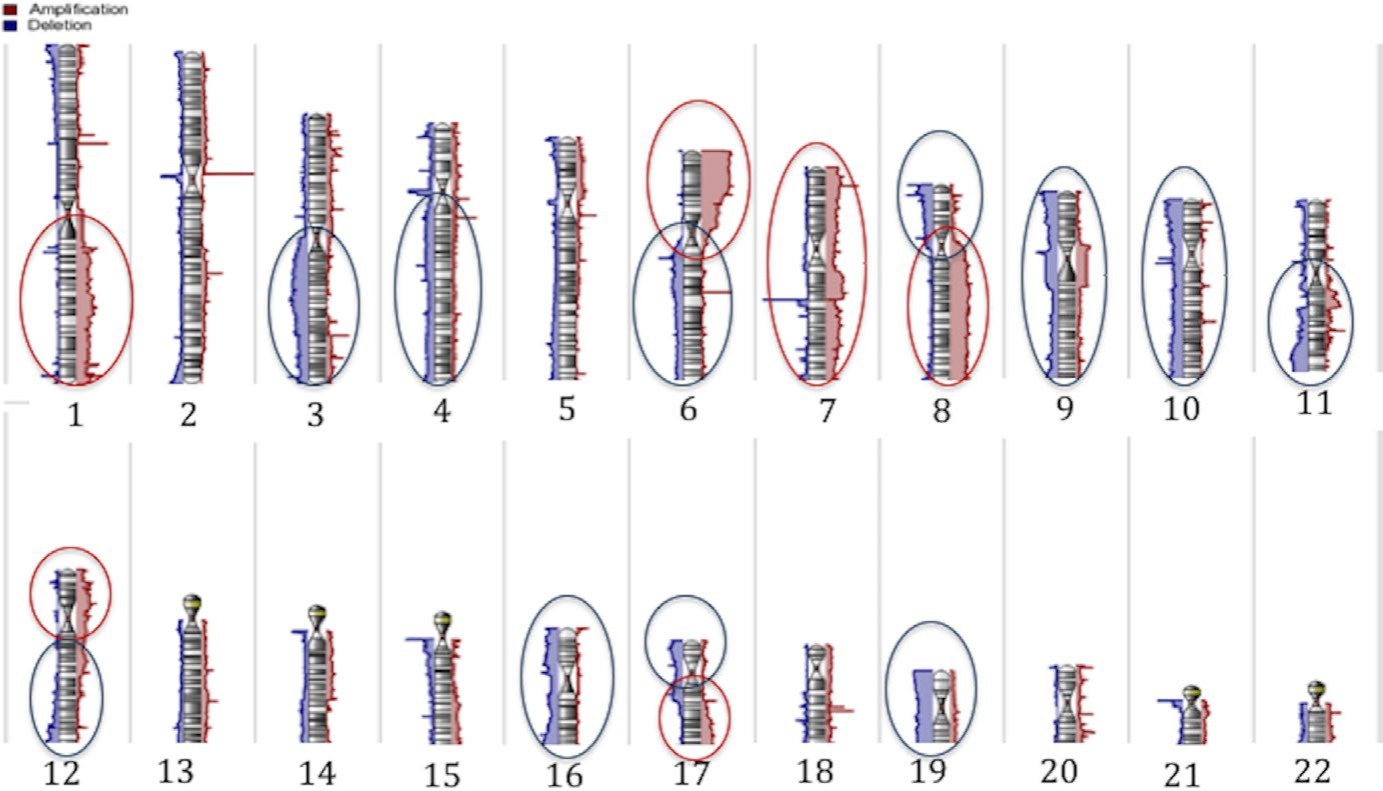
Karyogram of 59 CoM samples. Gross amplifications are shown in red and deletions in blue ovals

SNP genotype call rates were 91%–95% (mean, 94%). In these 59 samples, gross amplifications were observed in chr 1q, 6p, 7, 8q, 12p, and 17q, and deletions in chr 3q, 4q, 6p, 8p, 9, 10, 11q, 12q, 16, 17p, 19, and 22, Figure 1. A total of 25,103 gene‐specific CNAs were detected. No amplified or deleted CNAs were exclusive to the locally recurrent CoMs, whereas 1,069 were exclusive to the primary CoM. The most frequent amplifications in the whole cohort were observed on chr 6p21.31‐25.3 occurring in 76% of the 59 CoM. Specifically, amplifications in the Histone Cluster 1 group (6p22.2) were seen in up to 61%. The most frequent deletion noted was of the *ASNS* (7q21.3) gene, in up to 76% of the samples. Supporting Information Tables S2 and S3 list CNAs gains and losses present in ≥40% of the 59 samples. Following Bonferroni correction for multiple testing, no statistically significant correlations were detected between the common CNAs and clinical or histomorphological tumor features, Table 1.

**Table 1:** Univariate correlation of CoM common copy number alterations with clinical and histological features

Focus/region affected	CNA^a^	Clinical features				Histological features							Relation to mutation	
		Bulbar tumor	Caruncular tumor	Palpebral tumor	Metastasis	Extension to lateral surgical margin	Extension to deep surgical margin	Presence of lymphatic invasion	Presence of vascular invasion	Increasing tumor thickness	Mitotic count ≤5 versus >5/5HPF	Presence of epithelioid cells	*BRAF* mutation	*NRAS* mutation
Chr 6p21.32‐25.3	Amplification	1	0.61	0.64	1	0.05	0.45	1	0.7	0.74	0.75	0.41	0.65	1
*ETV1* (7p21.2)	Amplification	0.38	0.56	0.35	1	0.49	0.62	0.12	0.92	0.17	0.33	0.15	0.78	0.07
*SMAD4* (18q21.2)	Amplification	0.24	0.69	0.05	0.11	0.9	0.65	0.03	0.21	0.99	0.25	1	0.21	1
Chr 1q42.2‐43	Amplification	0.97	0.98	0.18	0.49	0.09	0.98	0.53	0.45	0.23	0.39	0.62	0.29	1
*CDK6 *(7q21.2)	Amplification	0.09	0.33	0.01	0.33	0.67	0.37	0.05	0.76	0.05	0.66	0.64	0.28	0.03
Chr 8q13.3	Amplification	0.26	0.51	0.04	1	0.91	0.37	0.84	0.42	0.13	0.09	0.64	0.5	0.67
*ASNS* (7q21.3)	Deletion	1	1	0.75	0.69	0.02	0.41	0.25	0.12	0.38	0.02	0.21	0.46	0.57
Chr 8p23.1‐23.3	Deletion	0.43	0.6	0.65	0.88	0.44	0.93	0.5	0.27	0.85	0.69	0.61	0.39	1
Chr 21p11.2‐11.1	Deletion	0.97	0.98	0.82	0.52	0.23	0.09	0.92	0.19	0.18	0.33	1	0.38	0.42
Chr 10q11.21‐11.22	Deletion	0.47	0.5	0.75	0.74	0.08	0.98	0.94	0.54	0.96	0.97	0.63	0.02	0.08
Significantly deleted tumor suppressor genes in Work Package 2														
Chr 10q24.32‐24.33 (*SUFU* and *NEURL1*)	Deletion	0.33	0.21	0.55	0.02	0.95	0.86	0.02	0.09	0.005	0.16	1	0.08	0.31
Chr 10q25.1‐25.2 (*PDCD4*)	Deletion	0.33	0.21	0.55	0.02	0.95	0.86	0.02	0.09	0.005	0.13	1	0.08	0.31
Chr 10q26.13‐26.2 (*C10orf90*)	Deletion	0.52	0.24	0.34	0.04	0.7	0.65	0.009	0.19	0.02	0.27	1	0.02	0.31

a Copy number alteration; *p* values: Mann–Whitney for correlation with thickness, otherwise Pearson's chi‐square or Fisher's exact as appropriate (2‐tailed significance).

**Figure 2:**
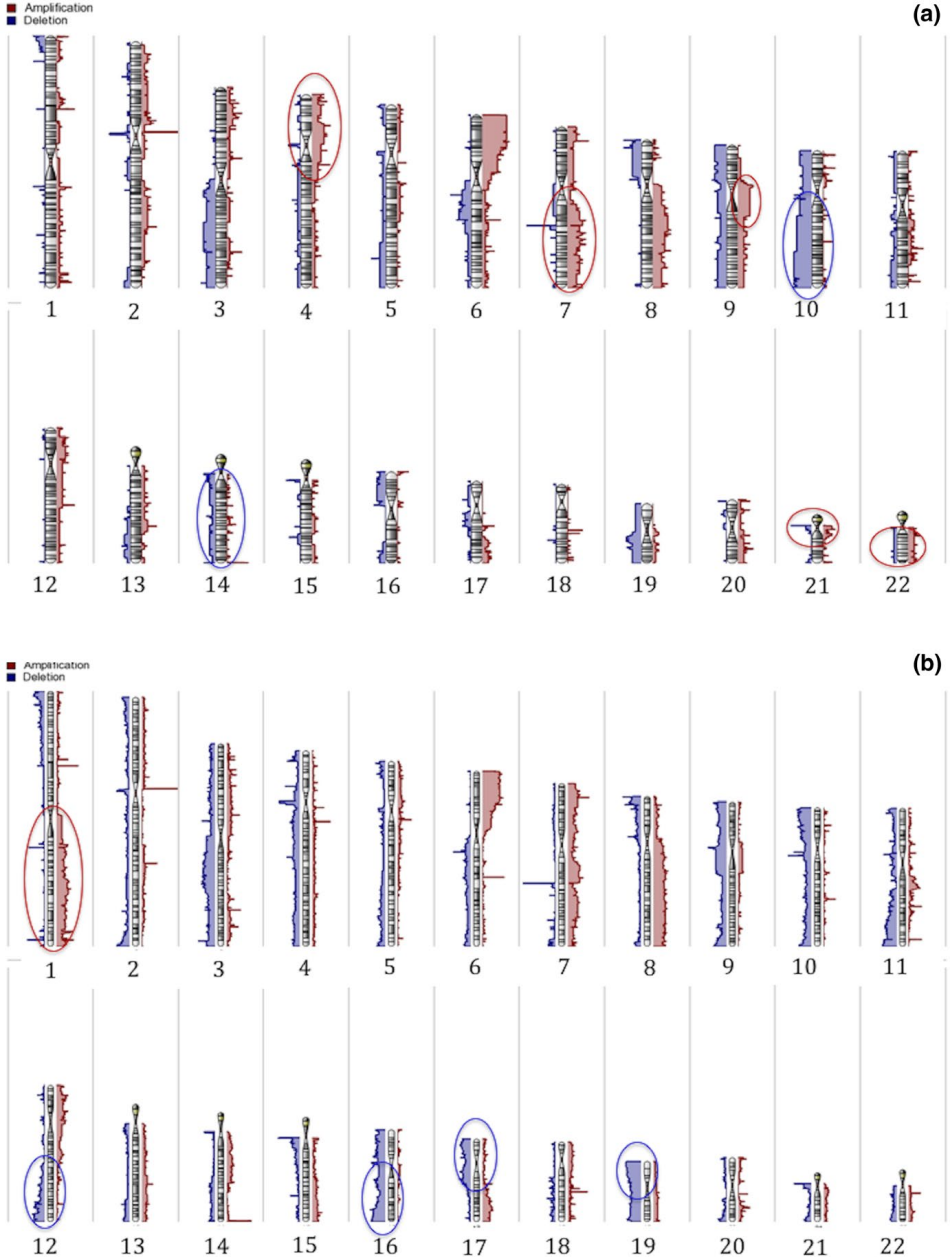
Karyogram of (a) 12 CoM that metastasized—CoMMET+ and (b) 35 without metastasis—CoMMET−. Gross differences in amplifications are marked in red and deletions in blue ovals

Forty‐seven (80%) of the 59 patients had ≥3.4 years of follow‐up, which (as above) is the literature‐reported median time to systemic metastasis (Tuomaala & Kivela, 2004). Twelve (26%) patients developed metastasis (CoMMET+), and 35 (74%) were metastasis‐free by study closure (CoMMET−). Karyograms of the gross alterations of the two groups are depicted in Figure 2. On detailed investigation, 25,099 gene‐specific CNAs were detected in CoMMET+ compared to 25,202 in CoMMET−. Of these CNAs, 25,017 were common to both groups while 82 were unique to CoMMET+ and 185 to CoMMET−, Supporting Information Tables S4 and S5.

Copy‐neutral allelic imbalances (CNAI), also known as allelic homozygosity or regions of homozygosity (ROH), corresponded to genes and regions identified as having normal/diploid copy number (CN) and were the most common imbalances, whereas homo‐ and hemizygous allelic imbalances (AIs) were less frequent. Homozygous deletions of 10q26.3 occurred in 50% (*n* = 6) of CoMMET+ group.

### 3.3 Identification of CNAs associated with CoM metastatic risk

**Figure 3:**
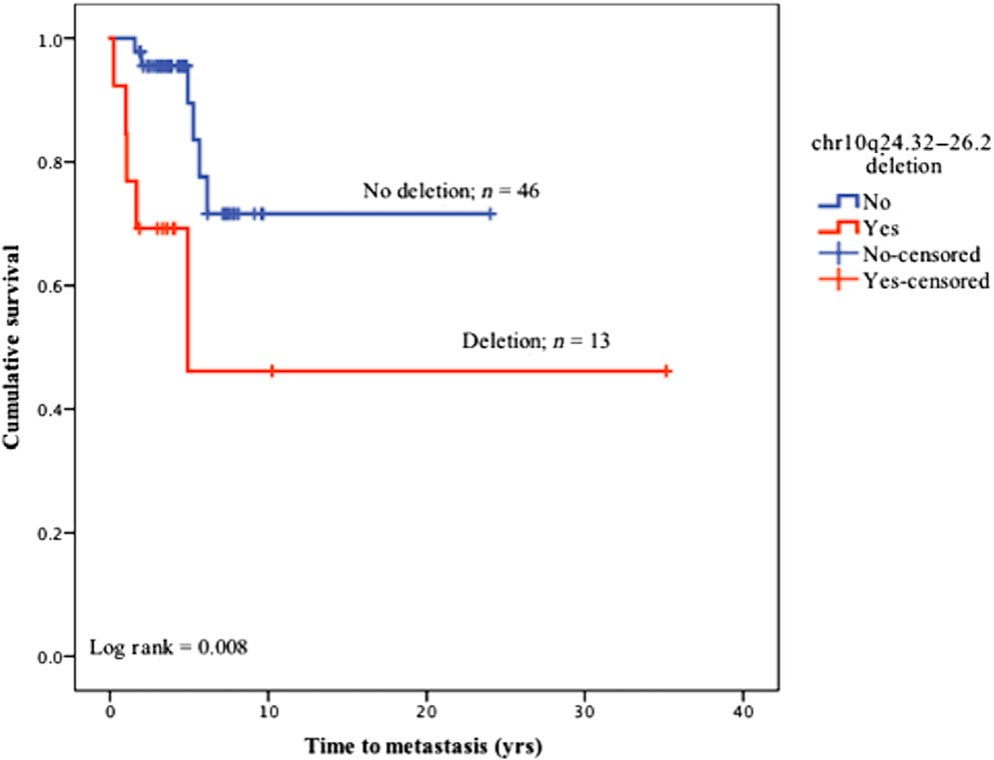
KM curve of chr 10q24.32‐26.2 deletion in relation to metastasis performed on 59 CoM patients. Tumors having the deleted 10q region were significantly associated with reduced metastasis‐free survival

No oncogenes or TSGs were identified in the CNAs exclusive to CoMMET+ group. In comparison, the TSG *PLPP5* (8p11.23) was exclusive to CoMMET− samples but was amplified in 3 and deleted in a further 3 samples (Fisher's exact, *p* = 1), so was not analyzed further. By comparing the CNAs present in at least 50% of tumors in the two groups, four TSGs on chr 10q24.32‐26.2 were significantly deleted in CoMMET+ tumors in contrast to CoMMET− group. These were *NEURL1*,* SUFU*,* PDCD4*, and *C10orf90* (Fisher's exact, *p* ≤ 0.05). Kaplan–Meier survival curves on the 59 patients estimated this regional deletion to be significantly associated with lower metastasis‐free survival (log‐rank, *p* = 0.008, Figure 3), and this was confirmed in those 47 patients with FU ≥3.4 years (logistic regression, *p* = 0.005; hazard ratio: 15). In addition, 10q24.32‐26.2 deletions were strongly associated with lymphatic invasion (Pearson's chi‐square, *p* ≤ 0.02) and increasing tumor thickness (range, 0.25–19 mm; median, 5.1 mm; mean, 6.8; mode, 3) compared with normal CN CoM (range, 0.25–17 mm; median, 2 mm; mean, 3.2; mode, 0.5), Table 1. These significant correlations were maintained after Bonferroni correction for multiple testing. No correlation was found between chr 10q deletions and CoM location, surgical margin involvement with tumor cells, vascular infiltration, mitotic count, or presence of epithelioid cell, Table 1.

### 3.4 Correlation between CNAs and mutation status

Eighteen (34%) of the 53 CoM were *BRAF*‐mt and 35 (66%) were *BRAF* wt, in keeping with published data. The mutations detected were as follows: V600E/EC in 15 samples (83%), V600K in two (11%), and V600R in one tumor (5%). *NRAS* mutations were detected in 6 (13%) of the 45 tested samples, of which five (83%) were in codon 61 and one (17%) in codon 12; 39 (87%) tumors were *NRAS* wt. *BRAF* and *NRAS* mutations were mutually exclusive except for the single tumor harboring the *BRAF* V600R mutation, which was also *NRAS* c61 mutant. After excluding the sample with concomitant *BRAF* and *NRAS* mutations, the 44 tumors in which both *BRAF* and *NRAS* mutations were analyzed, the mutation status was as follows: *BRAF*‐mt/*NRAS* wt in 14 (31%) tumors, *BRAF* wt/*NRAS*‐mt in 5 (11%), and wt for both genes 25 (56%) tumors.

By comparing only the *BRAF*‐mt and *NRAS*‐mt tumors, no clinical or histological features were significantly associated (*p* > 0.05) with the mutation status (Table 2). Significant regional amplifications were observed on chr 17q in *NRAS*‐mt tumors (Fisher's exact, *p* = 0.01) but did not include known oncogenes. In contrast, significant regional deletions were noted on chr 10q in *BRAF*‐mt tumors (Fisher's exact, *p* ≤ 0.03), Supporting Information Table S6. The deleted TSGs on chr 10q11.21‐23.31 (*RASSF4,* *C10orf99,* and *PTEN*) and 10q26.11‐26.3 (*DMBT1,* *C10orf90*) significantly correlated with *BRAF* mutation (Fisher's exact, *p* ≤ 0.04), Supporting Information Table S6.

**Table 2:** Univariate correlation of conjunctival melanoma mutation status with clinical and histological features

	BRAF‐mutant CoM (n = 14)	NRAS‐mutant CoM (n = 5)	p‐Value difference between the 2 groups	Test used
Clinical data				
Gender	Female: 4	Female: 3	0.25	Fisher's exact
	Male: 10	Male: 2		
Age at presentation (years)	37–92 (median and mode: 58)	59–88 (median 65.5)	0.45	1‐way ANOVA
CoM‐related metastasis	7	Nil	0.1	Fisher's exact
Time to metastasis (years)	0.25–6.1 (mean: 8.1; median: 2)	Not applicable		
Metastatic death	5	Not applicable		
Time to metastatic death (years)	1.8–7 (mean: 4.9; median: 6.7)	Not applicable		
Location	Bulbar: 8	Bulbar: 5	0.26	Fisher's exact
	Palpebral: 1			
	Caruncular: 5			
T stage^a^	T1a: 5; T1b: 1; T1c: 1; T1d: 1	T1a: 2; T1b: 2; T1c: 1	0.07	1‐way ANOVA
	T2b: 1; T2c: 2; T2d: 3			
Histopathological data				
Mitotic count/5HPF	0–11 (median: 1.5; mode: 1)	0–3 (median and mode: 1)	0.83	1‐way ANOVA
Thickness (mm)	0.25–18 (median: 2.1)	0.25–4.3 (median: 1.6)	0.35	1‐way ANOVA
Presence of epithelioid cells	Yes: 14	Yes: 5	1	Fisher's exact
	No: 0	No: 0		
Lymphatic invasion	Yes: 8	Yes: 1	0.3	Fisher's exact
	No: 6	No: 4		
Vascular invasion	Yes: 6	Yes: 2	1	Fisher's exact
	No: 8	No: 3		
Involvement of deep surgical margin	Yes: 6	Yes: 3	0.63	Fisher's exact
	No: 8	No: 2		
Involvement of lateral surgical margin	Yes: 9	Yes: 4	1	Fisher's exact
	No: 5	No: 1		
pT stage^a^	pT1a: 1; pT1b: 2; pT1c: 4	pT1a: 1; pT1b: 3; pT1c: 1	0.051	1‐way ANOVA
	pT2a: 1; pT2b: 1; pT2c: 4			
	pT3: 1			

a Staging according to the 7th AJCC staging system for conjunctival melanom.

No significant correlation was detected between *BRAF* or *NRAS* gene dosage and their mutation frequency.

### 3.5 Examining the effect of deletions on protein expression in selected samples

IHC protein expression localization and intensity scoring of the four significantly deleted TSGs in CoMs that metastasized showed:

1. NEURL1 expression was assessable in the 15 examined CoMs. Cytoplasmic expression scores ranged between 6 and 12 (mean; 8.4 and median; 8) in five CoMs with *NEURL1* deletion, and 4 to 12 (mean and median: 8) in ten tumors with diploid CN. PM staining was also present in 10% of cells of a single tumor with diploid CN. Staining was additionally detected in 5%–25% (mean, 11%; and median, 5%) of the nuclei in 4 (80%) CoMs with the deletion and 0%–90% (mean, 34.5%; and median, 5%) of the nuclei in 7 (70%) samples with diploid CN, representing the nucleocytoplasmic shuttling (Timmusk, Palm, Belluardo, Mudo, & Neuman, 2002).
2. PDCD4 expression was assessable in the 14 examined CoMs. Concurrent cytoplasmic and nuclear expression was detected in 3 (60%) of the five samples with deletion and 3 (33%) of the nine normal CN tumors, reflecting the nucleocytoplasmic shuttling (Bohm et al., 2003). Isolated nuclear expression was present in 2 (40%) and 4 (44%) CoMs with the respective gene deletion and diploid CN, respectively. No expression was detected in a further 2 (22%) diploid CN tumors. The cytoplasmic expression scores ranged between 0 and 4 (mean, 1.8; and median, 1) for the deleted *PDCD4* tumors, and 0–12 (mean, 2.7; and median, 0) in the normal CN CoMs. Nuclear staining was detected in 5%–95% (mean, 47%; and median, 30%) and 0%–80% (mean, 38%; and median, 50%) of the nuclei in the tumors with *PDCD4* deletion and diploid CN, respectively.
3. SUFU expression was assessable in the 14 examined CoMs. Expression was detected in the nuclei of 4 (80%) of 5 CoMs with the respective gene deletion (range, 0%–90%; mean, 70%; median, 80%) and all 9 samples with diploid CN (range, 10%–100%; mean, 72%; median, 80%).
4. C10orf90 expression was assessable in the 13 examined CoMs. Cytoplasmic staining was identified in 100% of the samples. Expression staining scores ranged between 4 and 12 (mean and median: 8) for the CoMs with *C10orf90* deletion and 3–12 (mean, 7.4; and median, 8) for the diploid CN tumors. Weak expression was also detected in 5% of the nuclei of a single sample with the gene deletion.

**Figure 4:**
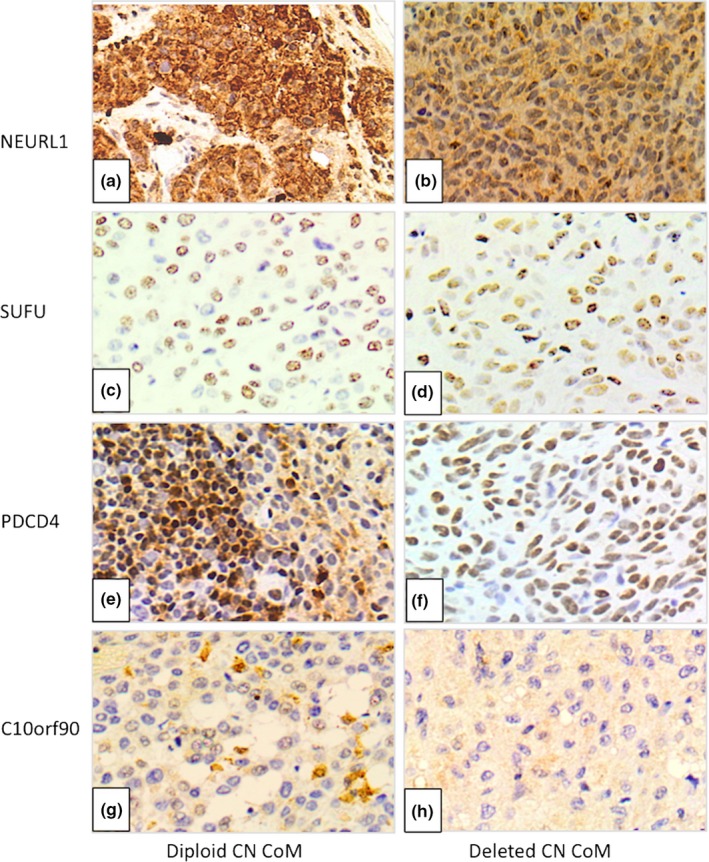
Representative immunohistochemistry micrographs of CoM tissue stained for NEURL1, SUFU, PDCD4, and C10orf90 proteins. NEURL1 nucleocytoplasmic and plasma membrane localization in normal (a) and nucleocytoplasmic only in deleted (b) copy number (CN) CoM. SUFU nuclear localization in normal (c) and deleted (d) CN CoM. PDCD4 nucleocytoplasmic localization in normal (e) and nuclear only in deleted (f) CN CoM. C10orf90 cytoplasmic localization in both normal (g) and deleted (h) CN CoM

Figure 4 shows representative IHC micrographs of CoM with normal and deleted CN stained for the four proteins.

For all proteins, the difference between the means of scores in normal and deleted CN tumors was not statistically significant (independent *t* test, *p* > 0.05); hence, there was no effect of the deleted CNAs on the respective protein expression, Supporting Information Table S7. This could be explained by the hemizygous deletion of the TSGs, and, therefore, in the presence of one functional allele protein expression would still be detected, and/or the differing binding sites of the antibodies. Alternatively, deletions caused by mutations and not detectable by SNP arrays could be the culprit and, therefore, prospective genome‐sequencing platforms should elucidate such events.

## 4 DISCUSSION

This is the first study to date to characterize, in depth and in such a large clinically well‐defined cohort, genome‐wide CNAs, their differential frequencies, and relationship to clinico‐histomorphological tumor features, *BRAF*/*NRAS* mutation, and clinical outcomes using high‐resolution SNP array genotyping technology.

Amplifications of 6p21‐25 were found in up to 61% of CoM in our cohort. Regional chr 6p amplification was previously identified by our group and others (Griewank et al., 2013; Lake et al., 2011; Swaminathan et al., 2017), and has also been documented in CM (Höglund et al., 2004). The Histone Cluster 1 (6p22.2) was the most common amplification in our study, which is implicated in various cancers and is thought to impact epigenetic and post‐transcriptional modification (King, Waxman, & Stauss, 2008), but was not associated with the clinical outcome in the current study.

In this study, we also identified novel regional and arm chromosomal losses on 9q, 16p, 17p, and 19, and more specifically of *ASNS* (7q21.3) focal deletion detected in 76% of CoM. ASNS expression is regarded as an important biomarker for therapeutic outcome in cancers. Hematological malignancies with *ASNS* deletions respond favorably to L‐asparaginase chemotherapeutics and rarely relapse (Bertuccio et al., 2017), whereas its low expression in colon carcinoma of patients undergoing chemoradiotherapy is associated with poor survival and inferior chemotherapeutic response (Lin et al., 2014). In our study, *ASNS* did not correlate with the clinical outcome of our patients; however, there is scope to examine its role in the management of the ocular tumor or its secondaries.

Most intriguing in this study are the detected 10q deletions in CoM and their correlation with metastatic risk, of which 10q26.3 was also previously described by our group (Lake et al., 2011). Our work has shown that regional deletions of chr 10q24.32‐26.2, and of the TSGs *NEURL1*, *SUFU*, *PDCD4*, and *C10orf90*, are significantly implicated in decreased metastasis‐free survival. While we did not observe this association at the protein level as assessed by IHC, this may be due to the variable hemizygous nature of the deletions and/or the differing binding sites of the antibodies. Importantly, however, the distal chr 10 losses were often linked with tumor thickness and the presence of lymphatic invasion, both recognized predictors of metastasis. In addition, our results corroborate the association between chr 10q deletions and *BRAF*‐mt CoMs, including the *PTEN* locus previously reported (Griewank et al., 2013). These are well‐recognized alterations in CM with implications on the oncogenic PI3K pathway inactivation and targeted therapy application (Isshiki, Elder, Guerry, & Linnenbach, 1993), and, therefore, they are potentially pertinent to CoM druggable targets.

Broad regional chromosomal alterations were characteristic of CoM rather than single gene aberrations. It remains unclear whether such regional anomalies are “bystander” alterations subsequent to single gene loci or non‐random events where the oncogenes and TSGs in the affected regions play roles at different stages of oncogenesis (Kwong & Chin, 2014). Irrespective of the cause, if such regional and multi‐gene involvement is maintained within metastatic CoMs, then, ideally, concomitant systemic therapies could be designed for CoM as suggested in CM (Carlson et al., 2017). There is no current clear guideline for the use of targeted therapeutics in metastatic CoM. There are limited anecdotal reports of potential inhibitory success on CoM cell lines and patients with Vemurafenib ± DaBRAFenib (*BRAF* V600E inhibitors), *MEK*, *AKT*, or *PD‐1* immune checkpoint inhibitors (Cao et al., 2017; Ford et al., 2017; Maleka, Astrom, Bystrom, & Ullenhag, 2016; Pinto Torres, Andre, Gouveia, Costa, & Passos, 2017; Riechardt et al., 2015; Sagiv et al., 2018). Notably, one of two patients with *BRAF‐*mt CoM treated with a *BRAF* inhibitor in our institution responded well to treatment (unpublished). In the absence of recognized therapeutic options for metastatic CoM and with extensive evidence from cutaneous melanoma (with which CoM shares many characteristics), *BRAF* and *PD‐1* inhibitors provide rational current treatment options.

Our work is the first to examine the role of AIs in CoM. Interestingly, normal CN of most regions was factual ROH, which are relevant to inactivating mutated TSGs or activating oncogenic mutations that can arise during tumorigenesis (O'Keefe, McDevitt, & Maciejewski, 2010). More interesting are the homozygous deletions of 10q26.3 in 50% of metastasizing CoMs, which could explain the gene deletions on 10q26.3 in most metastatic tumor tissue identified by Lake et al. (2011). Further projects elaborating on the AIs, including sequencing, could reveal their role in primary tumor evolution and metastatic progression, and, perhaps, risk stratification or treatment response as described in other cancers, such as colorectal and bladder carcinoma (Primdahl et al., 2002; Van Loo et al., 2010).

The current study also provides further evidence supporting the similarities between CoM and skin melanoma. *BRAF* and* NRAS* mutation frequencies, and their mutual exclusivity, were comparable to CM (Curtin et al., 2005; Davies et al., 2002), and in agreement with previous CoM reports (Griewank et al., 2013; Larsen et al., 2016; Scholz et al., 2018). We did not detect a correlation between the mutation status and clinical or histopathological features when compared to previous studies (Griewank et al., 2013; Larsen et al., 2016). This could be explained by the difference in analytical methods.

We acknowledge the limitations of our study. First, with variable treatment regimens and follow‐up times of the participating patients, it was not possible to assess the relationship between genetic alterations and CoM local recurrence. Nevertheless, the increased risk of metastasis following local CoM recurrence is well‐known (Damato & Coupland, 2009). Long‐term collaborative studies are essential to determine how radiation and topical chemotherapeutic methods could affect CoM biology. This should be undertaken as a future project when more samples become available. Second, the relation between chr 10q deletions and metastatic death was difficult to assess because of low number of events and the survival of some patients with metastases in our cohort. A prospective reanalysis of chr 10 and any related fatalities is warranted. Third, *BRAF* and *NRAS* mutations were tested by different techniques, and, as a result, their rarer mutations might have been missed in a small number of cases. In addition, it is possible that tumors that were both *BRAF* wt and *NRAS* wt harbor *NF1 *or other *RAS *mutations, which were reported only after the completion of the current work (Scholz et al., 2018). A follow‐up study that incorporates all known CoM mutations is now needed to understand their significance in CoM pathogenesis.

In summary, we here present the most comprehensive profile of CNAs in a clinically well‐defined CoM cohort to date and identified potential markers for metastatic risk and prognostication. The ultimate challenge remains to apply our current knowledge in the future development of prognostic models and effective therapeutics in metastatic CoM, as has been achieved in part with skin melanoma.
