# Supplementary material for: Conjunctival melanoma copy number alterations and correlation with mutation status, tumor features, and clinical outcome
Source: Pigment Cell Melanoma Res. 2019 Feb 19;32(4):564–75. doi: 10.1111/pcmr.12767 (PMC6849808; doi:10.1111/pcmr.12767)
Supplement: Supplementary file 1 [file PCMR-32-564-s001.docx]

Supplementary tables

Supplementary table 1. Demographics of 59 patients and their CoM studied with aSNP array

| **Patients' demographics** | **Sex** | Female: 30; Male: 29 |
| --- | --- | --- |
|  | **Age (yrs)** | Range, 25-92; (median & mode: 64) |
|  | **Ethnicity** | Caucasians: 56 Non-Caucasians: 3 |
|  | **Overall survival** | 0.92-35.42 yrs (mean, 5.1; median, 3.8) |
|  | **Duration of follow up (yrs)** | 1.7-26 (median, 3.5) |
| **Clinical data** | **Number of tumors investigated with SNP array** | Primary: 52 Locally recurrent: 7 |
|  | **Laterality** | Right: 30 Left: 29 |
|  | **Tumor location** | Bulbar only: 38 Palpebral: 13 Caruncular: 8 |
|  | **Tumor color** | Black-dark brown: 3 Dark brown: 32 Light brown: 2 Pink-red: 13 Mixed: 4 Yellow: 1 Unknown: 4 |
|  | **Tumor configuration** | Nodular: 47 Diffuse: 12 |
|  | **Number of tumors that metastasised** | 12 |
|  | **Organ of metastasis** | Orbit: 1 LN: 2 Systemic: 9 |
|  | **Time to metastasis** | 0.6-6.1 yrs (mean, 3.7; median, 3.5) |
|  | **Metastatic deaths** | 8 |
|  | **Time to metastatic deaths (yrs)** | 1.1-6.9 (mean, 5; median, 6.4) |
| **Histopathological data** | **Thickness** | Range, 0.25-19 mm; median, 2; mean, 3.8; mode, 0.5) |
|  | **Mitotic count** | Range, 0-28 mm; median, 2; mean, 4.1; mode, 1) |
|  | **Lateral surgical margin clearance** | Yes: 29; No: 30 |
|  | **Deep surgical margin clearance** | Yes: 32; No: 25  Not recorded: 2 |
|  | **Vascular invasion** | Yes: 21; No: 38 |
|  | **Lymphatic invasion** | Yes: 31; No: 48 |
|  | **Presence of epithelioid cells** | Yes: 55; No: 4 |
|  | **Presence of epithelioid cells** | Yes: 55; No: 4 |

Supplementary table 2. Common amplifications detected in ≥40% of 59 CoM by SNP array

| **Transcript chromosome** | **Gene Symbol** | **Cytoband** | **Total Amplifications no. (%)** | **Amplification Average Copy Number** |
| --- | --- | --- | --- | --- |
| 6 | *HIST1H1T* | 6p22.2 | 36 (61) | 2.91703 |
| 6 | *HIST1H2BC* | 6p22.2 | 36 (61) | 3.00785 |
| 6 | *HIST1H2AC* | 6p22.2 | 36 (61) | 3.00785 |
| 6 | *HIST1H1E* | 6p22.2 | 36 (61) | 3.00785 |
| 6 | *HIST1H2BD* | 6p22.2 | 36 (61) | 3.00785 |
| 6 | *HIST1H2BE* | 6p22.2 | 36 (61) | 3.00785 |
| 6 | *HIST1H4D* | 6p22.2 | 36 (61) | 3.00785 |
| 6 | *HIST1H3D* | 6p22.2 | 36 (61) | 3.00785 |
| 6 | *HIST1H2AD* | 6p22.2 | 36 (61) | 3.00785 |
| 6 | *HIST1H2BF* | 6p22.2 | 36 (61) | 3.00785 |
| 6 | *HIST1H4E* | 6p22.2 | 36 (61) | 3.00785 |
| 6 | *HIST1H2BG* | 6p22.2 | 36 (61) | 3.00785 |
| 6 | *HIST1H2AE* | 6p22.2 | 36 (61) | 3.00785 |
| 6 | *HIST1H3E* | 6p22.2 | 36 (61) | 3.00785 |
| 6 | *HIST1H1D* | 6p22.2 | 36 (61) | 3.00785 |
| 6 | *HIST1H4F* | 6p22.2 | 36 (61) | 3.00785 |
| 6 | *HIST1H4G* | 6p22.2 | 36 (61) | 3.00785 |
| 6 | *HIST1H3F* | 6p22.2 | 36 (61) | 3.00785 |
| 6 | *HIST1H2BH* | 6p22.2 | 36 (61) | 3.00785 |
| 6 | *HIST1H3G* | 6p22.2 | 36 (61) | 3.00785 |
| 6 | *HIST1H2BI* | 6p22.2 | 36 (61) | 3.00785 |
| 6 | *HIST1H4H* | 6p22.2 | 36 (61) | 3.00785 |
| 7 | *ETV1* | 7p21.2 | 35 (59) | 4.449 |
| 6 | *SERPINB6* | 6p25.2 | 34 (58) | 3.24424 |
| 6 | *LINC01011* | 6p25.2 | 34 (58) | 3.24424 |
| 6 | *NQO2* | 6p25.2 | 34 (58) | 3.13654 |
| 6 | *HFE* | 6p22.2 | 34 (58) | 2.88134 |
| 6 | *HLA-DRA* | 6p21.32 | 33 (56) | 3.28372 |
| 6 | *EXOC2* | 6p25.3 | 32 (54) | 2.78794 |
| 6 | *HUS1B* | 6p25.3 | 32 (54) | 2.78794 |
| 6 | *LOC285768* | 6p25.3 - 6p25.2 | 32 (54) | 2.71675 |
| 6 | *FOXQ1* | 6p25.3 - 6p25.2 | 32 (54) | 2.71675 |
| 6 | *FOXF2* | 6p25.3 - 6p25.2 | 32 (54) | 2.71675 |
| 6 | *MIR6720* | 6p25.3 - 6p25.2 | 32 (54) | 2.71675 |
| 6 | *FOXCUT* | 6p25.3 - 6p25.2 | 32 (54) | 2.71675 |
| 6 | *FOXC1* | 6p25.3 - 6p25.2 | 32 (54) | 2.71675 |
| 6 | *GMDS* | 6p25.3 - 6p25.2 | 32 (54) | 2.71675 |
| 6 | *GMDS-AS1* | 6p25.3 - 6p25.2 | 32 (54) | 2.71675 |
| 6 | *LINC01600* | 6p25.2 | 32 (54) | 2.69681 |
| 6 | *MYLK4* | 6p25.2 | 32 (54) | 2.69681 |
| 6 | *WRNIP1* | 6p25.2 | 32 (54) | 2.69681 |
| 6 | *SERPINB1* | 6p25.2 | 32 (54) | 2.89063 |
| 6 | *MIR4645* | 6p25.2 | 32 (54) | 2.89063 |
| 6 | *SERPINB9P1* | 6p25.2 | 32 (54) | 2.89063 |
| 6 | *LOC101927730* | 6p25.2 | 32 (54) | 2.89063 |
| 6 | *SERPINB9* | 6p25.2 | 32 (54) | 2.89063 |
| 6 | *RIPK1* | 6p25.2 | 32 (54) | 2.81272 |
| 6 | *HLA-DRB5* | 6p21.32 | 32 (54) | 3.33331 |
| 6 | *HLA-DRB6* | 6p21.32 | 32 (54) | 3.33331 |
| 6 | *HLA-DRB1* | 6p21.32 | 32 (54) | 3.33331 |
| 6 | *LOC101927691* | 6p25.3 | 31 (53) | 2.70915 |
| 6 | *BPHL* | 6p25.2 | 31 (53) | 2.68429 |
| 6 | *TUBB2A* | 6p25.2 | 31 (53) | 2.68429 |
| 6 | *LOC100507194* | 6p25.2 | 31 (53) | 2.68429 |
| 6 | *TUBB2B* | 6p25.2 | 31 (53) | 2.68429 |
| 6 | *PSMG4* | 6p25.2 | 31 (53) | 2.68429 |
| 6 | *SLC22A23* | 6p25.2 | 31 (53) | 2.68429 |
| 6 | *PXDC1* | 6p25.2 - 6p25.1 | 31 (53) | 2.68408 |
| 6 | *FAM50B* | 6p25.2 - 6p25.1 | 31 (53) | 2.68408 |
| 6 | *PRPF4B* | 6p25.2 - 6p25.1 | 31 (53) | 2.68408 |
| 6 | *FAM217A* | 6p25.2 - 6p25.1 | 31 (53) | 2.68408 |
| 6 | *C6orf201* | 6p25.2 - 6p25.1 | 31 (53) | 2.68408 |
| 6 | *ECI2* | 6p25.2 - 6p25.1 | 31 (53) | 2.68408 |
| 6 | *LOC100507506* | 6p25.2 - 6p25.1 | 31 (53) | 2.68408 |
| 6 | *LOC102724096* | 6p25.2 - 6p25.1 | 31 (53) | 2.68408 |
| 6 | *MIR7641-2* | 6p25.2 - 6p25.1 | 31 (53) | 2.68408 |
| 6 | *KU-MEL-3* | 6p25.2 - 6p25.1 | 31 (53) | 2.68408 |
| 6 | *CDYL* | 6p25.2 - 6p25.1 | 31 (53) | 2.68408 |
| 6 | *RPP40* | 6p25.2 - 6p25.1 | 31 (53) | 2.68408 |
| 6 | *LOC100129461* | 6p25.2 - 6p25.1 | 31 (53) | 2.68408 |
| 6 | *PPP1R3G* | 6p25.2 - 6p25.1 | 31 (53) | 2.68408 |
| 6 | *LYRM4* | 6p25.2 - 6p25.1 | 31 (53) | 2.68408 |
| 6 | *MIR3691* | 6p25.2 - 6p25.1 | 31 (53) | 2.68408 |
| 6 | *FARS2* | 6p25.2 - 6p25.1 | 31 (53) | 2.68408 |
| 6 | *LOC101927972* | 6p25.2 - 6p25.1 | 31 (53) | 2.68408 |
| 6 | *LOC101927950* | 6p25.2 - 6p25.1 | 31 (53) | 2.68408 |
| 6 | *RREB1* | 6p25.1 - 6p24.3 | 31 (53) | 2.68934 |
| 6 | *SSR1* | 6p25.1 - 6p24.3 | 31 (53) | 2.68934 |
| 6 | *CAGE1* | 6p25.1 - 6p24.3 | 31 (53) | 2.68934 |
| 6 | *RIOK1* | 6p25.1 - 6p24.3 | 31 (53) | 2.68934 |
| 6 | *DSP* | 6p24.3 | 31 (53) | 2.68408 |
| 6 | *SNRNP48* | 6p24.3 | 31 (53) | 2.68408 |
| 6 | *BMP6* | 6p24.3 | 31 (53) | 2.68408 |
| 6 | *BLOC1S5-TXNDC5* | 6p24.3 | 31 (53) | 2.68408 |
| 6 | *TXNDC5* | 6p24.3 | 31 (53) | 2.68408 |
| 6 | *PIP5K1P1* | 6p24.3 | 31 (53) | 2.68408 |
| 6 | *BLOC1S5* | 6p24.3 | 31 (53) | 2.68408 |
| 6 | *EEF1E1-BLOC1S5* | 6p24.3 | 31 (53) | 2.68408 |
| 6 | *EEF1E1* | 6p24.3 | 31 (53) | 2.68408 |
| 6 | *SCARNA27* | 6p24.3 | 31 (53) | 2.68408 |
| 6 | *SLC35B3* | 6p24.3 | 31 (53) | 2.68408 |
| 6 | *LOC100506207* | 6p24.3 | 31 (53) | 2.68408 |
| 6 | *HULC* | 6p24.3 | 31 (53) | 2.68408 |
| 6 | *TFAP2A* | 6p24.3 - 6p24.2 | 31 (53) | 2.69508 |
| 6 | *TFAP2A-AS1* | 6p24.3 - 6p24.2 | 31 (53) | 2.69508 |
| 6 | *LINC00518* | 6p24.3 - 6p24.2 | 31 (53) | 2.69508 |
| 6 | *MIR5689* | 6p24.3 - 6p24.2 | 31 (53) | 2.69508 |
| 6 | *GCNT2* | 6p24.3 - 6p24.2 | 31 (53) | 2.69508 |
| 6 | *C6orf52* | 6p24.3 - 6p24.2 | 31 (53) | 2.69508 |
| 6 | *PAK1IP1* | 6p24.3 - 6p24.2 | 31 (53) | 2.69508 |
| 6 | *TMEM14C* | 6p24.3 - 6p24.2 | 31 (53) | 2.69508 |
| 6 | *TMEM14B* | 6p24.3 - 6p24.2 | 31 (53) | 2.69508 |
| 6 | *MAK* | 6p24.3 - 6p24.2 | 31 (53) | 2.69508 |
| 6 | *GCM2* | 6p24.3 - 6p24.2 | 31 (53) | 2.69508 |
| 6 | *SYCP2L* | 6p24.3 - 6p24.2 | 31 (53) | 2.69508 |
| 6 | *LOC101928191* | 6p24.3 - 6p24.2 | 31 (53) | 2.69508 |
| 6 | *ELOVL2* | 6p24.3 - 6p24.2 | 31 (53) | 2.69508 |
| 6 | *ELOVL2-AS1* | 6p24.3 - 6p24.2 | 31 (53) | 2.69508 |
| 6 | *SMIM13* | 6p24.3 - 6p24.2 | 31 (53) | 2.69508 |
| 6 | *ERVFRD-1* | 6p24.3 - 6p24.2 | 31 (53) | 2.69508 |
| 6 | *NEDD9* | 6p24.2 | 31 (53) | 2.68709 |
| 6 | *JARID2* | 6p22.3 | 31 (53) | 2.909 |
| 6 | *JARID2-AS1* | 6p22.3 | 31 (53) | 2.909 |
| 6 | *DTNBP1* | 6p22.3 | 31 (53) | 2.84708 |
| 6 | *MYLIP* | 6p22.3 | 31 (53) | 2.77182 |
| 6 | *MIR4639* | 6p22.3 | 31 (53) | 2.77182 |
| 6 | *GMPR* | 6p22.3 | 31 (53) | 2.77182 |
| 6 | *ATXN1* | 6p22.3 | 31 (53) | 2.77182 |
| 6 | *STMND1* | 6p22.3 | 31 (53) | 2.77182 |
| 6 | *CAP2* | 6p22.3 | 31 (53) | 2.75552 |
| 6 | *LOC101928491* | 6p22.3 | 31 (53) | 2.75552 |
| 6 | *FAM8A1* | 6p22.3 | 31 (53) | 2.75552 |
| 6 | *NUP153* | 6p22.3 | 31 (53) | 2.75552 |
| 6 | *KIF13A* | 6p22.3 | 31 (53) | 2.75552 |
| 6 | *NHLRC1* | 6p22.3 | 31 (53) | 2.75552 |
| 6 | *TPMT* | 6p22.3 | 31 (53) | 2.75552 |
| 6 | *KDM1B* | 6p22.3 | 31 (53) | 2.75552 |
| 6 | *DEK* | 6p22.3 | 31 (53) | 2.75552 |
| 6 | *RNF144B* | 6p22.3 | 31 (53) | 2.75552 |
| 6 | *MIR548A1* | 6p22.3 | 31 (53) | 2.75552 |
| 6 | *LOC101928519* | 6p22.3 | 31 (53) | 2.75552 |
| 6 | *ID4* | 6p22.3 | 31 (53) | 2.75552 |
| 6 | *MBOAT1* | 6p22.3 | 31 (53) | 2.75552 |
| 6 | *E2F3* | 6p22.3 | 31 (53) | 2.75552 |
| 6 | *CDKAL1* | 6p22.3 | 31 (53) | 2.75552 |
| 6 | *LINC00581* | 6p22.3 | 31 (53) | 2.75552 |
| 6 | *SOX4* | 6p22.3 | 31 (53) | 2.75552 |
| 6 | *CASC15* | 6p22.3 | 31 (53) | 2.75552 |
| 6 | *NBAT1* | 6p22.3 | 31 (53) | 2.83147 |
| 6 | *PRL* | 6p22.3 | 31 (53) | 2.83147 |
| 6 | *HDGFL1* | 6p22.3 | 31 (53) | 2.76696 |
| 6 | *DCDC2* | 6p22.3 - 6p22.2 | 31 (53) | 2.70103 |
| 6 | *KAAG1* | 6p22.3 - 6p22.2 | 31 (53) | 2.70103 |
| 6 | *MRS2* | 6p22.3 - 6p22.2 | 31 (53) | 2.70103 |
| 6 | *GPLD1* | 6p22.3 - 6p22.2 | 31 (53) | 2.70103 |
| 6 | *ALDH5A1* | 6p22.3 - 6p22.2 | 31 (53) | 2.70103 |
| 6 | *KIAA0319* | 6p22.3 - 6p22.2 | 31 (53) | 2.70103 |
| 6 | *TDP2* | 6p22.3 - 6p22.2 | 31 (53) | 2.70103 |
| 6 | *ACOT13* | 6p22.3 - 6p22.2 | 31 (53) | 2.70103 |
| 6 | *C6orf62* | 6p22.3 - 6p22.2 | 31 (53) | 2.70103 |
| 6 | *GMNN* | 6p22.3 - 6p22.2 | 31 (53) | 2.70103 |
| 6 | *C6orf229* | 6p22.3 - 6p22.2 | 31 (53) | 2.70103 |
| 6 | *FAM65B* | 6p22.3 - 6p22.2 | 31 (53) | 2.70103 |
| 6 | *CMAHP* | 6p22.3 - 6p22.2 | 31 (53) | 2.70103 |
| 6 | *LOC101928663* | 6p22.3 - 6p22.2 | 31 (53) | 2.70103 |
| 6 | *LRRC16A* | 6p22.3 - 6p22.2 | 31 (53) | 2.70103 |
| 6 | *SCGN* | 6p22.3 - 6p22.2 | 31 (53) | 2.70103 |
| 6 | *BTN3A2* | 6p22.2 | 31 (53) | 2.79211 |
| 6 | *BTN2A2* | 6p22.2 | 31 (53) | 2.79211 |
| 6 | *BTN3A1* | 6p22.2 | 31 (53) | 2.79211 |
| 6 | *BTN2A3P* | 6p22.2 | 31 (53) | 2.79211 |
| 6 | *BTN3A3* | 6p22.2 | 31 (53) | 2.79211 |
| 6 | *BTN2A1* | 6p22.2 | 31 (53) | 2.79211 |
| 6 | *LOC285819* | 6p22.2 | 31 (53) | 2.79211 |
| 6 | *BTN1A1* | 6p22.2 | 31 (53) | 2.79211 |
| 6 | *HCG11* | 6p22.2 | 31 (53) | 2.79211 |
| 6 | *HMGN4* | 6p22.2 | 31 (53) | 2.79211 |
| 6 | *ABT1* | 6p22.2 | 31 (53) | 2.70602 |
| 6 | *ZNF322* | 6p22.2 | 31 (53) | 2.66724 |
| 18 | *SMAD4* | 18q21.2 | 30 (51) | 3.51732 |
| 6 | *IRF4* | 6p25.3 | 30 (51) | 2.71602 |
| 6 | *NRN1* | 6p25.1 | 30 (51) | 2.67536 |
| 6 | *F13A1* | 6p25.1 | 30 (51) | 2.67536 |
| 6 | *MIR5683* | 6p25.1 | 30 (51) | 2.67536 |
| 6 | *LY86-AS1* | 6p25.1 | 30 (51) | 2.67536 |
| 6 | *LY86* | 6p25.1 | 30 (51) | 2.67536 |
| 6 | *TMEM170B* | 6p24.2 - 6p24.1 | 30 (51) | 2.68586 |
| 6 | *ADTRP* | 6p24.2 - 6p24.1 | 30 (51) | 2.68586 |
| 6 | *LOC101928253* | 6p24.1 | 30 (51) | 2.68739 |
| 6 | *HIVEP1* | 6p24.1 | 30 (51) | 2.68739 |
| 6 | *PHACTR1* | 6p24.1 - 6p23 | 30 (51) | 2.67849 |
| 6 | *LOC100130357* | 6p24.1 - 6p23 | 30 (51) | 2.67849 |
| 6 | *TBC1D7* | 6p24.1 - 6p23 | 30 (51) | 2.67849 |
| 6 | *GFOD1* | 6p24.1 - 6p23 | 30 (51) | 2.67849 |
| 6 | *SIRT5* | 6p24.1 - 6p23 | 30 (51) | 2.67849 |
| 6 | *NOL7* | 6p24.1 - 6p23 | 30 (51) | 2.67849 |
| 6 | *RANBP9* | 6p24.1 - 6p23 | 30 (51) | 2.67849 |
| 6 | *MCUR1* | 6p24.1 - 6p23 | 30 (51) | 2.67849 |
| 6 | *RNF182* | 6p24.1 - 6p23 | 30 (51) | 2.67849 |
| 6 | *CD83* | 6p24.1 - 6p23 | 30 (51) | 2.67849 |
| 6 | *LINC01108* | 6p24.1 - 6p23 | 30 (51) | 2.67849 |
| 6 | *NRSN1* | 6p22.3 | 30 (51) | 2.6943 |
| 6 | *SLC17A4* | 6p22.2 | 30 (51) | 2.69513 |
| 6 | *SLC17A1* | 6p22.2 | 30 (51) | 2.69513 |
| 6 | *SLC17A3* | 6p22.2 | 30 (51) | 2.69513 |
| 6 | *SLC17A2* | 6p22.2 | 30 (51) | 2.69513 |
| 6 | *TRIM38* | 6p22.2 | 30 (51) | 2.69513 |
| 6 | *GUSBP2* | 6p22.2 | 30 (51) | 2.73746 |
| 6 | *LINC00240* | 6p22.2 - 6p22.1 | 30 (51) | 2.66748 |
| 6 | *LOC100270746* | 6p22.2 - 6p22.1 | 30 (51) | 2.66748 |
| 6 | *HIST cluster 1* | 6p22.2 - 6p22.1 | 30 (51) | 2.66748 |
| 6 | *MIR3143* | 6p22.2 - 6p22.1 | 30 (51) | 2.66748 |
| 6 | *PRSS16* | 6p22.2 - 6p22.1 | 30 (51) | 2.66748 |
| 6 | *POM121L2* | 6p22.2 - 6p22.1 | 30 (51) | 2.66748 |
| 6 | *VN1R10P* | 6p22.2 - 6p22.1 | 30 (51) | 2.66748 |
| 6 | *ZNF204P* | 6p22.2 - 6p22.1 | 30 (51) | 2.66748 |
| 6 | *ZNF391* | 6p22.2 - 6p22.1 | 30 (51) | 2.66748 |
| 6 | *ZNF184* | 6p22.2 - 6p22.1 | 30 (51) | 2.66748 |
| 6 | *LINC01012* | 6p22.2 - 6p22.1 | 30 (51) | 2.66748 |
| 6 | *LOC100131289* | 6p22.2 - 6p22.1 | 30 (51) | 2.66748 |
| 6 | *OR2B2* | 6p22.2 - 6p22.1 | 30 (51) | 2.66748 |
| 6 | *OR2B6* | 6p22.2 - 6p22.1 | 30 (51) | 2.66748 |
| 6 | *ZNF165* | 6p22.2 - 6p22.1 | 30 (51) | 2.66748 |
| 6 | *ZSCAN12P1* | 6p22.2 - 6p22.1 | 30 (51) | 2.66748 |
| 6 | *ZSCAN16-AS1* | 6p22.2 - 6p22.1 | 30 (51) | 2.66748 |
| 6 | *ZSCAN16* | 6p22.2 - 6p22.1 | 30 (51) | 2.66748 |
| 6 | *ZKSCAN8* | 6p22.2 - 6p22.1 | 30 (51) | 2.66748 |
| 6 | *ZNF192P1* | 6p22.2 - 6p22.1 | 30 (51) | 2.66748 |
| 6 | *TOB2P1* | 6p22.2 - 6p22.1 | 30 (51) | 2.66748 |
| 6 | *ZSCAN9* | 6p22.2 - 6p22.1 | 30 (51) | 2.66748 |
| 6 | *ZKSCAN4* | 6p22.2 - 6p22.1 | 30 (51) | 2.66748 |
| 6 | *NKAPL* | 6p22.2 - 6p22.1 | 30 (51) | 2.66748 |
| 6 | *ZSCAN26* | 6p22.2 - 6p22.1 | 30 (51) | 2.66748 |
| 6 | *PGBD1* | 6p22.2 - 6p22.1 | 30 (51) | 2.66748 |
| 6 | *ZSCAN31* | 6p22.2 - 6p22.1 | 30 (51) | 2.66748 |
| 6 | *ZKSCAN3* | 6p22.2 - 6p22.1 | 30 (51) | 2.66748 |
| 6 | *ZSCAN12* | 6p22.2 - 6p22.1 | 30 (51) | 2.66748 |
| 6 | *ZSCAN23* | 6p22.2 - 6p22.1 | 30 (51) | 2.66748 |
| 6 | *GPX6* | 6p22.2 - 6p22.1 | 30 (51) | 2.66748 |
| 6 | *GPX5* | 6p22.2 - 6p22.1 | 30 (51) | 2.66748 |
| 6 | *ZBED9* | 6p22.2 - 6p22.1 | 30 (51) | 2.66748 |
| 6 | *LOC401242* | 6p22.2 - 6p22.1 | 30 (51) | 2.66748 |
| 6 | *HCG14* | 6p22.2 - 6p22.1 | 30 (51) | 2.66748 |
| 6 | *TRIM27* | 6p22.2 - 6p22.1 | 30 (51) | 2.66748 |
| 6 | *LINC01556* | 6p22.2 - 6p22.1 | 30 (51) | 2.66748 |
| 6 | *ZNF311* | 6p22.2 - 6p22.1 | 30 (51) | 2.66748 |
| 6 | *LOC100129636* | 6p22.2 - 6p22.1 | 30 (51) | 2.66748 |
| 6 | *OR2W1* | 6p22.2 - 6p22.1 | 30 (51) | 2.66748 |
| 6 | *OR2B3* | 6p22.2 - 6p22.1 | 30 (51) | 2.66748 |
| 6 | *OR2J3* | 6p22.2 - 6p22.1 | 30 (51) | 2.66748 |
| 6 | *OR2J2* | 6p22.2 - 6p22.1 | 30 (51) | 2.66748 |
| 6 | *OR14J1* | 6p22.2 - 6p22.1 | 30 (51) | 2.66748 |
| 6 | *OR5V1* | 6p22.2 - 6p22.1 | 30 (51) | 2.66748 |
| 6 | *OR12D3* | 6p22.2 - 6p22.1 | 30 (51) | 2.66748 |
| 6 | *OR12D2* | 6p22.2 - 6p22.1 | 30 (51) | 2.66748 |
| 6 | *OR11A1* | 6p22.2 - 6p22.1 | 30 (51) | 2.66748 |
| 6 | *OR10C1* | 6p22.2 - 6p22.1 | 30 (51) | 2.66748 |
| 6 | *OR2H1* | 6p22.2 - 6p22.1 | 30 (51) | 2.66748 |
| 6 | *MAS1L* | 6p22.2 - 6p22.1 | 30 (51) | 2.66748 |
| 6 | *LINC01015* | 6p22.2 - 6p22.1 | 30 (51) | 2.66748 |
| 6 | *UBD* | 6p22.2 - 6p22.1 | 30 (51) | 2.66748 |
| 6 | *SNORD32B* | 6p22.2 - 6p22.1 | 30 (51) | 2.66748 |
| 6 | *OR2H2* | 6p22.2 - 6p22.1 | 30 (51) | 2.66748 |
| 6 | *GABBR1* | 6p22.2 - 6p22.1 | 30 (51) | 2.66748 |
| 6 | *MOG* | 6p22.2 - 6p22.1 | 30 (51) | 2.66748 |
| 6 | *ZFP57* | 6p22.2 - 6p22.1 | 30 (51) | 2.66748 |
| 6 | *HLA-F* | 6p22.2 - 6p22.1 | 30 (51) | 2.66748 |
| 6 | *HLA-F-AS1* | 6p22.2 - 6p22.1 | 30 (51) | 2.66748 |
| 6 | *IFITM4P* | 6p22.2 - 6p22.1 | 30 (51) | 2.66748 |
| 6 | *HCG4* | 6p22.2 - 6p22.1 | 30 (51) | 2.66748 |
| 6 | *LOC554223* | 6p22.2 - 6p22.1 | 30 (51) | 2.66748 |
| 6 | *HLA-G* | 6p22.2 - 6p22.1 | 30 (51) | 2.66748 |
| 6 | *RBM24* | 6p22.3 | 29 (49) | 2.73639 |
| 6 | *HCG4B* | 6p22.1 | 29 (49) | 2.84399 |
| 6 | *HLA-A* | 6p22.1 | 29 (49) | 2.84399 |
| 6 | *HCG9* | 6p22.1 | 29 (49) | 2.84399 |
| 6 | *ZNRD1-AS1* | 6p22.1 - 6p21.33 | 29 (49) | 2.6631 |
| 6 | *HCG8* | 6p22.1 - 6p21.33 | 29 (49) | 2.6631 |
| 6 | *ZNRD1* | 6p22.1 - 6p21.33 | 29 (49) | 2.6631 |
| 6 | *PPP1R11* | 6p22.1 - 6p21.33 | 29 (49) | 2.6631 |
| 6 | *RNF39* | 6p22.1 - 6p21.33 | 29 (49) | 2.6631 |
| 6 | *TRIM31* | 6p22.1 - 6p21.33 | 29 (49) | 2.6631 |
| 6 | *TRIM31-AS1* | 6p22.1 - 6p21.33 | 29 (49) | 2.6631 |
| 6 | *TRIM40* | 6p22.1 - 6p21.33 | 29 (49) | 2.6631 |
| 6 | *TRIM10* | 6p22.1 - 6p21.33 | 29 (49) | 2.6631 |
| 6 | *TRIM15* | 6p22.1 - 6p21.33 | 29 (49) | 2.6631 |
| 6 | *TRIM26* | 6p22.1 - 6p21.33 | 29 (49) | 2.6631 |
| 6 | *HCG17* | 6p22.1 - 6p21.33 | 29 (49) | 2.6631 |
| 6 | *MIR6891* | 6p22.1 - 6p21.33 | 29 (49) | 2.6631 |
| 6 | *HCG18* | 6p22.1 - 6p21.33 | 29 (49) | 2.6631 |
| 6 | *TRIM39* | 6p22.1 - 6p21.33 | 29 (49) | 2.6631 |
| 6 | *TRIM39-RPP21* | 6p22.1 - 6p21.33 | 29 (49) | 2.6631 |
| 6 | *RPP21* | 6p22.1 - 6p21.33 | 29 (49) | 2.6631 |
| 6 | *HLA-E* | 6p21.33 | 29 (49) | 2.73811 |
| 6 | *GNL1* | 6p21.33 | 29 (49) | 2.83787 |
| 6 | *PRR3* | 6p21.33 | 29 (49) | 2.83787 |
| 6 | *ABCF1* | 6p21.33 | 29 (49) | 2.83787 |
| 6 | *MIR877* | 6p21.33 | 29 (49) | 2.83787 |
| 6 | *PPP1R10* | 6p21.33 | 29 (49) | 2.83787 |
| 6 | *MRPS18B* | 6p21.33 | 29 (49) | 2.83787 |
| 6 | *ATAT1* | 6p21.33 | 29 (49) | 2.83787 |
| 6 | *C6orf136* | 6p21.33 | 29 (49) | 2.83787 |
| 6 | *DHX16* | 6p21.33 | 29 (49) | 2.83787 |
| 6 | *PPP1R18* | 6p21.33 | 29 (49) | 2.83787 |
| 6 | *NRM* | 6p21.33 | 29 (49) | 2.83787 |
| 6 | *MDC1* | 6p21.33 | 29 (49) | 2.83787 |
| 6 | *TUBB* | 6p21.33 | 29 (49) | 2.83787 |
| 6 | *FLOT1* | 6p21.33 | 29 (49) | 2.83787 |
| 6 | *IER3* | 6p21.33 | 29 (49) | 2.83787 |
| 6 | *LINC00243* | 6p21.33 | 29 (49) | 2.83787 |
| 6 | *DDR1* | 6p21.33 | 29 (49) | 2.70804 |
| 6 | *MIR4640* | 6p21.33 | 29 (49) | 2.70804 |
| 6 | *GTF2H4* | 6p21.33 | 29 (49) | 2.70804 |
| 6 | *VARS2* | 6p21.33 | 29 (49) | 2.70804 |
| 6 | *SFTA2* | 6p21.33 | 29 (49) | 2.65727 |
| 6 | *DPCR1* | 6p21.33 | 29 (49) | 2.65727 |
| 6 | *MUC21* | 6p21.33 | 29 (49) | 2.65727 |
| 6 | *MUC22* | 6p21.33 | 29 (49) | 2.65727 |
| 6 | *HCG22* | 6p21.33 | 29 (49) | 2.65727 |
| 6 | *C6orf15* | 6p21.33 | 29 (49) | 2.65727 |
| 6 | *PSORS1C1* | 6p21.33 | 29 (49) | 2.65727 |
| 6 | *CDSN* | 6p21.33 | 29 (49) | 2.65727 |
| 6 | *PSORS1C2* | 6p21.33 | 29 (49) | 2.65727 |
| 6 | *CCHCR1* | 6p21.33 | 29 (49) | 2.65727 |
| 6 | *TCF19* | 6p21.33 | 29 (49) | 2.65727 |
| 6 | *POU5F1* | 6p21.33 | 29 (49) | 2.65727 |
| 6 | *PSORS1C3* | 6p21.33 | 29 (49) | 2.65727 |
| 6 | *HCG27* | 6p21.33 | 29 (49) | 2.65727 |
| 6 | *HLA-DQA1* | 6p21.32 | 29 (49) | 3.1364 |
| 4 | *UGT2B17* | 4q13.2 | 28 (47) | 3.40941 |
| 6 | *EDN1* | 6p24.1 | 28 (47) | 2.63442 |
| 6 | *RNU6-48P* | 6p24.1 | 28 (47) | 2.63442 |
| 1 | *LINC01347* | 1q43 | 27 (48) | 3.0699 |
| 1 | *CEP170* | 1q43 | 27 (48) | 3.0699 |
| 1 | *SDCCAG8* | 1q43 | 27 (48) | 3.0699 |
| 6 | *HLA-C* | 6p21.33 | 27 (48) | 2.6488 |
| 6 | *HLA-B* | 6p21.33 | 27 (48) | 2.6488 |
| 6 | *MICA* | 6p21.33 | 27 (48) | 2.6488 |
| 6 | *MICB* | 6p21.33 | 27 (48) | 2.64763 |
| 6 | *MCCD1* | 6p21.33 - 6p21.32 | 27 (48) | 2.64763 |
| 6 | *ATP6V1G2-DDX39B* | 6p21.33 - 6p21.32 | 27 (48) | 2.64763 |
| 6 | *DDX39B* | 6p21.33 - 6p21.32 | 27 (48) | 2.64763 |
| 6 | *SNORD117* | 6p21.33 - 6p21.32 | 27 (48) | 2.64763 |
| 6 | *SNORD84* | 6p21.33 - 6p21.32 | 27 (48) | 2.64763 |
| 6 | *ATP6V1G2* | 6p21.33 - 6p21.32 | 27 (48) | 2.64763 |
| 6 | *NFKBIL1* | 6p21.33 - 6p21.32 | 27 (48) | 2.64763 |
| 6 | *LTA* | 6p21.33 - 6p21.32 | 27 (48) | 2.64763 |
| 6 | *TNF* | 6p21.33 - 6p21.32 | 27 (48) | 2.64763 |
| 6 | *LTB* | 6p21.33 - 6p21.32 | 27 (48) | 2.64763 |
| 6 | *LST1* | 6p21.33 - 6p21.32 | 27 (48) | 2.64763 |
| 6 | *NCR3* | 6p21.33 - 6p21.32 | 27 (48) | 2.64763 |
| 6 | *AIF1* | 6p21.33 - 6p21.32 | 27 (48) | 2.64763 |
| 6 | *PRRC2A* | 6p21.33 - 6p21.32 | 27 (48) | 2.64763 |
| 6 | *SNORA38* | 6p21.33 - 6p21.32 | 27 (48) | 2.64763 |
| 6 | *MIR6832* | 6p21.33 - 6p21.32 | 27 (48) | 2.64763 |
| 6 | *BAG6* | 6p21.33 - 6p21.32 | 27 (48) | 2.64763 |
| 6 | *APOM* | 6p21.33 - 6p21.32 | 27 (48) | 2.64763 |
| 6 | *C6orf47* | 6p21.33 - 6p21.32 | 27 (48) | 2.64763 |
| 6 | *GPANK1* | 6p21.33 - 6p21.32 | 27 (48) | 2.64763 |
| 6 | *CSNK2B* | 6p21.33 - 6p21.32 | 27 (48) | 2.64763 |
| 6 | *LY6G5B* | 6p21.33 - 6p21.32 | 27 (48) | 2.64763 |
| 6 | *LY6G5C* | 6p21.33 - 6p21.32 | 27 (48) | 2.64763 |
| 6 | *ABHD16A* | 6p21.33 - 6p21.32 | 27 (48) | 2.64763 |
| 6 | *MIR4646* | 6p21.33 - 6p21.32 | 27 (48) | 2.64763 |
| 6 | *LY6G6F* | 6p21.33 - 6p21.32 | 27 (48) | 2.64763 |
| 6 | *LY6G6E* | 6p21.33 - 6p21.32 | 27 (48) | 2.64763 |
| 6 | *LY6G6D* | 6p21.33 - 6p21.32 | 27 (48) | 2.64763 |
| 6 | *LY6G6C* | 6p21.33 - 6p21.32 | 27 (48) | 2.64763 |
| 6 | *C6orf25* | 6p21.33 - 6p21.32 | 27 (48) | 2.64763 |
| 6 | *DDAH2* | 6p21.33 - 6p21.32 | 27 (48) | 2.64763 |
| 6 | *CLIC1* | 6p21.33 - 6p21.32 | 27 (48) | 2.64763 |
| 6 | *MSH5* | 6p21.33 - 6p21.32 | 27 (48) | 2.64763 |
| 6 | *MSH5-SAPCD1* | 6p21.33 - 6p21.32 | 27 (48) | 2.64763 |
| 6 | *SAPCD1* | 6p21.33 - 6p21.32 | 27 (48) | 2.64763 |
| 6 | *SAPCD1-AS1* | 6p21.33 - 6p21.32 | 27 (48) | 2.64763 |
| 6 | *VWA7* | 6p21.33 - 6p21.32 | 27 (48) | 2.64763 |
| 6 | *VARS* | 6p21.33 - 6p21.32 | 27 (48) | 2.64763 |
| 6 | *LSM2* | 6p21.33 - 6p21.32 | 27 (48) | 2.64763 |
| 6 | *HSPA1L* | 6p21.33 - 6p21.32 | 27 (48) | 2.64763 |
| 6 | *HSPA1A* | 6p21.33 - 6p21.32 | 27 (48) | 2.64763 |
| 6 | *HSPA1B* | 6p21.33 - 6p21.32 | 27 (48) | 2.64763 |
| 6 | *C6orf48* | 6p21.33 - 6p21.32 | 27 (48) | 2.64763 |
| 6 | *SNORD48* | 6p21.33 - 6p21.32 | 27 (48) | 2.64763 |
| 6 | *SNORD52* | 6p21.33 - 6p21.32 | 27 (48) | 2.64763 |
| 6 | *NEU1* | 6p21.33 - 6p21.32 | 27 (48) | 2.64763 |
| 6 | *SLC44A4* | 6p21.33 - 6p21.32 | 27 (48) | 2.64763 |
| 6 | *EHMT2* | 6p21.33 - 6p21.32 | 27 (48) | 2.64763 |
| 6 | *C2* | 6p21.33 - 6p21.32 | 27 (48) | 2.64763 |
| 6 | *ZBTB12* | 6p21.33 - 6p21.32 | 27 (48) | 2.64763 |
| 6 | *C2-AS1* | 6p21.33 - 6p21.32 | 27 (48) | 2.64763 |
| 6 | *CFB* | 6p21.33 - 6p21.32 | 27 (48) | 2.64763 |
| 6 | *NELFE* | 6p21.33 - 6p21.32 | 27 (48) | 2.64763 |
| 6 | *MIR1236* | 6p21.33 - 6p21.32 | 27 (48) | 2.64763 |
| 6 | *SKIV2L* | 6p21.33 - 6p21.32 | 27 (48) | 2.64763 |
| 6 | *DXO* | 6p21.33 - 6p21.32 | 27 (48) | 2.64763 |
| 6 | *STK19* | 6p21.33 - 6p21.32 | 27 (48) | 2.64763 |
| 6 | *C4A* | 6p21.33 - 6p21.32 | 27 (48) | 2.64763 |
| 6 | *C4B* | 6p21.33 - 6p21.32 | 27 (48) | 2.64763 |
| 6 | *C4B_2* | 6p21.33 - 6p21.32 | 27 (48) | 2.64763 |
| 6 | *CYP21A2* | 6p21.33 - 6p21.32 | 27 (48) | 2.64763 |
| 6 | *CYP21A1P* | 6p21.33 - 6p21.32 | 27 (48) | 2.64763 |
| 6 | *TNXA* | 6p21.33 - 6p21.32 | 27 (48) | 2.64763 |
| 6 | *TNXB* | 6p21.33 - 6p21.32 | 27 (48) | 2.64763 |
| 6 | *ATF6B* | 6p21.33 - 6p21.32 | 27 (48) | 2.64763 |
| 6 | *FKBPL* | 6p21.33 - 6p21.32 | 27 (48) | 2.64763 |
| 6 | *PRRT1* | 6p21.33 - 6p21.32 | 27 (48) | 2.64763 |
| 6 | *LOC100507547* | 6p21.33 - 6p21.32 | 27 (48) | 2.64763 |
| 6 | *PPT2* | 6p21.33 - 6p21.32 | 27 (48) | 2.64763 |
| 6 | *PPT2-EGFL8* | 6p21.33 - 6p21.32 | 27 (48) | 2.64763 |
| 6 | *EGFL8* | 6p21.33 - 6p21.32 | 27 (48) | 2.64763 |
| 6 | *AGPAT1* | 6p21.33 - 6p21.32 | 27 (48) | 2.64763 |
| 6 | *MIR6721* | 6p21.33 - 6p21.32 | 27 (48) | 2.64763 |
| 6 | *RNF5* | 6p21.33 - 6p21.32 | 27 (48) | 2.64763 |
| 6 | *RNF5P1* | 6p21.33 - 6p21.32 | 27 (48) | 2.64763 |
| 6 | *MIR6833* | 6p21.33 - 6p21.32 | 27 (48) | 2.64763 |
| 6 | *AGER* | 6p21.33 - 6p21.32 | 27 (48) | 2.64763 |
| 6 | *PBX2* | 6p21.33 - 6p21.32 | 27 (48) | 2.64763 |
| 6 | *GPSM3* | 6p21.33 - 6p21.32 | 27 (48) | 2.64763 |
| 6 | *NOTCH4* | 6p21.33 - 6p21.32 | 27 (48) | 2.64763 |
| 6 | *C6orf10* | 6p21.32 | 27 (48) | 2.67621 |
| 6 | *HCG23* | 6p21.32 | 27 (48) | 2.67621 |
| 6 | *BTNL2* | 6p21.32 | 27 (48) | 2.67621 |
| 6 | *HCP5* | 6p21.33 | 26 (44) | 2.65728 |
| 6 | *HCG26* | 6p21.33 | 26 (44) | 2.65728 |
| 6 | *HLA-DQB1* | 6p21.32 | 26 (44) | 2.88009 |
| 6 | *TAP2* | 6p21.32 | 26 (44) | 2.6828 |
| 6 | *PSMB8* | 6p21.32 | 26 (44) | 2.6828 |
| 6 | *PSMB8-AS1* | 6p21.32 | 26 (44) | 2.6828 |
| 6 | *TAP1* | 6p21.32 | 26 (44) | 2.6828 |
| 6 | *PSMB9* | 6p21.32 | 26 (44) | 2.6828 |
| 6 | *LOC100294145* | 6p21.32 | 26 (44) | 2.6828 |
| 6 | *HLA-DMB* | 6p21.32 | 26 (44) | 2.6828 |
| 6 | *HLA-DMA* | 6p21.32 | 26 (44) | 2.6828 |
| 6 | *BRD2* | 6p21.32 | 26 (44) | 2.6828 |
| 6 | *HLA-DOA* | 6p21.32 | 26 (44) | 2.6828 |
| 6 | *HLA-DPA1* | 6p21.32 | 26 (44) | 2.6828 |
| 6 | *HLA-DPB1* | 6p21.32 | 26 (44) | 2.6828 |
| 1 | *TARBP1* | 1q42.2 - 1q42.3 | 25 (42) | 2.8937 |
| 1 | *LINC01354* | 1q42.2 - 1q42.3 | 25 (42) | 2.8937 |
| 1 | *IRF2BP2* | 1q42.2 - 1q42.3 | 25 (42) | 2.8937 |
| 1 | *LINC00184* | 1q42.2 - 1q42.3 | 25 (42) | 2.8937 |
| 6 | *LOC285766* | 6p25.3 | 25 (42) | 2.71926 |
| 6 | *HLA-DQA2* | 6p21.32 | 25 (42) | 2.69798 |
| 6 | *MIR3135B* | 6p21.32 | 25 (42) | 2.69798 |
| 6 | *HLA-DQB2* | 6p21.32 | 25 (42) | 2.69798 |
| 6 | *HLA-DOB* | 6p21.32 | 25 (42) | 2.69798 |
| 6 | *HLA-DPB2* | 6p21.32 - 6p21.31 | 25 (42) | 2.67006 |
| 6 | *COL11A2* | 6p21.32 - 6p21.31 | 25 (42) | 2.67006 |
| 6 | *RXRB* | 6p21.32 - 6p21.31 | 25 (42) | 2.67006 |
| 6 | *SLC39A7* | 6p21.32 - 6p21.31 | 25 (42) | 2.67006 |
| 6 | *HSD17B8* | 6p21.32 - 6p21.31 | 25 (42) | 2.67006 |
| 6 | *MIR219A1* | 6p21.32 - 6p21.31 | 25 (42) | 2.67006 |
| 6 | *RING1* | 6p21.32 - 6p21.31 | 25 (42) | 2.67006 |
| 6 | *HCG25* | 6p21.32 - 6p21.31 | 25 (42) | 2.67006 |
| 6 | *VPS52* | 6p21.32 - 6p21.31 | 25 (42) | 2.67006 |
| 6 | *RPS18* | 6p21.32 - 6p21.31 | 25 (42) | 2.67006 |
| 6 | *B3GALT4* | 6p21.32 - 6p21.31 | 25 (42) | 2.67006 |
| 6 | *WDR46* | 6p21.32 - 6p21.31 | 25 (42) | 2.67006 |
| 6 | *MIR6873* | 6p21.32 - 6p21.31 | 25 (42) | 2.67006 |
| 6 | *PFDN6* | 6p21.32 - 6p21.31 | 25 (42) | 2.67006 |
| 6 | *MIR6834* | 6p21.32 - 6p21.31 | 25 (42) | 2.67006 |
| 6 | *RGL2* | 6p21.32 - 6p21.31 | 25 (42) | 2.67006 |
| 6 | *TAPBP* | 6p21.32 - 6p21.31 | 25 (42) | 2.67006 |
| 6 | *ZBTB22* | 6p21.32 - 6p21.31 | 25 (42) | 2.67006 |
| 6 | *MIR1234* | 6p21.32 - 6p21.31 | 25 (42) | 2.67006 |
| 6 | *DAXX* | 6p21.32 - 6p21.31 | 25 (42) | 2.67006 |
| 6 | *KIFC1* | 6p21.32 - 6p21.31 | 25 (42) | 2.67006 |
| 6 | *PHF1* | 6p21.32 - 6p21.31 | 25 (42) | 2.67006 |
| 6 | *CUTA* | 6p21.32 - 6p21.31 | 25 (42) | 2.67006 |
| 6 | *SYNGAP1* | 6p21.32 - 6p21.31 | 25 (42) | 2.67006 |
| 6 | *MIR5004* | 6p21.32 - 6p21.31 | 25 (42) | 2.67006 |
| 6 | *ZBTB9* | 6p21.32 - 6p21.31 | 25 (42) | 2.67006 |
| 6 | *BAK1* | 6p21.32 - 6p21.31 | 25 (42) | 2.67006 |
| 6 | *GGNBP1* | 6p21.32 - 6p21.31 | 25 (42) | 2.67006 |
| 6 | *LINC00336* | 6p21.32 - 6p21.31 | 25 (42) | 2.67006 |
| 6 | *ITPR3* | 6p21.32 - 6p21.31 | 25 (42) | 2.67006 |
| 6 | *UQCC2* | 6p21.32 - 6p21.31 | 25 (42) | 2.67006 |
| 6 | *MIR3934* | 6p21.32 - 6p21.31 | 25 (42) | 2.67006 |
| 6 | *IP6K3* | 6p21.32 - 6p21.31 | 25 (42) | 2.67006 |
| 6 | *LEMD2* | 6p21.32 - 6p21.31 | 25 (42) | 2.67006 |
| 6 | *MLN* | 6p21.32 - 6p21.31 | 25 (42) | 2.67006 |
| 6 | *DUSP22* | 6p25.3 | 24 (41) | 2.73119 |
| 6 | *LINC01016* | 6p21.31 | 24 (41) | 2.67487 |
| 6 | *MIR7159* | 6p21.31 | 24 (41) | 2.67487 |
| 6 | *HMGA1* | 6p21.31 | 24 (41) | 2.71736 |
| 6 | *MIR6835* | 6p21.31 | 24 (41) | 2.71736 |
| 6 | *C6orf1* | 6p21.31 | 24 (41) | 2.71736 |
| 6 | *NUDT3* | 6p21.31 | 24 (41) | 2.71736 |
| 6 | *RPS10-NUDT3* | 6p21.31 | 24 (41) | 2.71736 |
| 6 | *RPS10* | 6p21.31 | 24 (41) | 2.71736 |
| 6 | *SPDEF* | 6p21.31 | 24 (41) | 2.68794 |
| 6 | *C6orf106* | 6p21.31 | 24 (41) | 2.68794 |
| 6 | *SNRPC* | 6p21.31 | 24 (41) | 2.68794 |
| 6 | *UHRF1BP1* | 6p21.31 | 24 (41) | 2.68794 |
| 6 | *TAF11* | 6p21.31 | 24 (41) | 2.68794 |
| 6 | *ANKS1A* | 6p21.31 | 24 (41) | 2.68794 |
| 6 | *TCP11* | 6p21.31 | 24 (41) | 2.68794 |
| 6 | *SCUBE3* | 6p21.31 | 24 (41) | 2.68794 |
| 7 | *CDK6* | 7q21.2 | 24 (41) | 3.52934 |
| 8 | *MSC* | 8q13.3 | 24 (41) | 3.05393 |
| 8 | *MSC-AS1* | 8q13.3 | 24 (41) | 3.05393 |

Supplementary table 3. Frequent deletions detected in ≥ 40% of the 59 CoM used in aSNP array

| **Transcript chromosome** | **Gene Symbol** | **Cytoband** | **Total Deletions no. (%)** | **Deletion Average Copy Number** |
| --- | --- | --- | --- | --- |
| 7 | *ASNS* | 7q21.3 | 45 (76) | 1.10255 |
| 8 | *TDRP* | 8p23.3 | 28 (47) | 1.26561 |
| 8 | *ZNF705B* | 8p23.1 | 28 (47) | 1.33413 |
| 8 | *FAM66E* | 8p23.1 | 28 (47) | 1.33413 |
| 8 | *USP17L8* | 8p23.1 | 28 (47) | 1.33413 |
| 8 | *USP17L3* | 8p23.1 | 28 (47) | 1.33413 |
| 8 | *MIR548I3* | 8p23.1 | 28 (47) | 1.33413 |
| 21 | *TPTE* | 21p11.2 - 21p11.1 | 27 (46) | 1.31933 |
| 21 | *BAGE2* | 21p11.2 - 21p11.1 | 27 (46) | 1.31933 |
| 21 | *BAGE3* | 21p11.2 - 21p11.1 | 27 (46) | 1.31933 |
| 21 | *BAGE4* | 21p11.2 - 21p11.1 | 27 (46) | 1.31933 |
| 21 | *BAGE5* | 21p11.2 - 21p11.1 | 27 (46) | 1.31933 |
| 21 | *BAGE* | 21p11.2 - 21p11.1 | 27 (46) | 1.31933 |
| 10 | *RET* | 10q11.21 | 26 (44) | 1.21259 |
| 10 | *GPRIN2* | 10q11.22 | 26 (44) | 1.27443 |

Supplementary table 4. Copy number alterations exclusive to 12 CoM that metastasized (CoMMET+)

| *AGPAT6* | *C17orf85* | *CCDC101* | *KIAA0226* | *LOC100130700* | *LOC102724862* | *LOC80078* | *PPAP2A* | *WAPAL* |
| --- | --- | --- | --- | --- | --- | --- | --- | --- |
| *AGPAT9* | *C1orf110* | *CCDC176* | *KIAA1407* | *LOC100506457* | *LOC152578* | *LOH12CR1* | *PPAP2B* | *WIBG* |
| *C10orf32* | *C1orf177* | *CCDC37* | *KIAA1467* | *LOC100507462* | *LOC286370* | *LPPR1* | *PPAP2C* |  |
| *C10orf32-ASMT* | *C20orf62* | *CCDC42B* | *KIAA2018* | *LOC101060321* | *LOC388849* | *LPPR2* | *PPAPDC1A* |  |
| *C11orf39* | *C4orf29* | *CCHE1* | *LINC00669* | *LOC101927081* | *LOC391003* | *LPPR3* | *PPAPDC1B* |  |
| *C12orf79* | *C6orf165* | *CRAMP1L* | *LINC00883* | *LOC101928223* | *LOC400736* | *LPPR4* | *PPAPDC2* |  |
| *C15orf26* | *C6orf7* | *FAM183B* | *LINC00925* | *LOC101928790* | *LOC645354* | *LPPR5* | *PPAPDC3* |  |
| *C15orf27* | *C9orf117* | *ITFG3* | *LINC01573* | *LOC101928989* | *LOC645382* | *LRRC48* | *SLMO1* |  |
| *C16orf93* | *C9orf156* | *KIAA0020* | *LINC01604* | *LOC101929983* | *LOC649324* | *MEF2BNB* | *SLMO2* |  |
| *C17orf59* | *C9orf171* | *KIAA0195* | *LINS* | *LOC102723859* | *LOC731424* | *NUPL1* | *SMIM11* |  |

Supplementary table 5. Copy number alterations exclusive to CoMs without metastasis at ≥ 3.4 years follow up

| *ABHD18* | *DRC3* | *LINC01052* | *LOC101927759* | *LUADT1* | *PLPP1* | *PYM1* | *SNORA89* | *SNORD141B* |
| --- | --- | --- | --- | --- | --- | --- | --- | --- |
| *ARF4-AS1* | *DUBR* | *LINC01078* | *LOC101928098* | *MINCR* | *PLPP2* | *RUBCN* | *SNORA90* | *SNORD142* |
| *BBOF1* | *FAM183BP* | *LINC01173* | *LOC101929208* | *MIR193BHG* | *PLPP3* | *SCARNA26A* | *SNORA91* | *SNORD143* |
| *BORCS5* | *FAM234A* | *LINC01297* | *LOC101929227* | *MIR34AHG* | *PLPP4* | *SCARNA26B* | *SNORA92* | *SNORD144* |
| *BORCS6* | *FAM234B* | *LINC01318* | *LOC101929726* | *MIR3681HG* | *PLPP5* | *SCARNA28* | *SNORA93* | *STT3A-AS1* |
| *BORCS7* | *GLTSCR2-AS1* | *LINC01381* | *LOC102724571* | *MIR3945HG* | *PLPP6* | *SGF29* | *SNORA94* | *TBC1D3E* |
| *BORCS7-ASMT* | *GPAT3* | *LINC01596* | *LOC102724652* | *MIR4290HG* | *PLPP7* | *SMIM11A* | *SNORA95* | *TBC1D3G* |
| *BORCS8* | *GPAT4* | *LINC01612* | *LOC105369532* | *MIR4300HG* | *PLPPR1* | *SMIM11B* | *SNORA98* | *TBC1D3I* |
| *CCDC188* | *GRPEL2-AS1* | *LINC01613* | *LOC105370333* | *MIR4307HG* | *PLPPR2* | *SNORA100* | *SNORA99* | *TCF4-AS1* |
| *CCDC189* | *GSTT1-AS1* | *LINC01614* | *LOC105372071* | *MIR4432HG* | *PLPPR3* | *SNORA103* | *SNORD128* | *TMEM14EP* |
| *CCDC190* | *GVQW2* | *LINC01615* | *LOC105375650* | *MIR9-3HG* | *PLPPR4* | *SNORA104* | *SNORD129* | *TMEM266* |
| *CCDC191* | *KCNK15-AS1* | *LINC01616* | *LOC105376331* | *MIR924HG* | *PLPPR5* | *SNORA105A* | *SNORD130* | *TMEM94* |
| *CCEPR* | *LCAL1* | *LINC01618* | *LOC105376575* | *NALT1* | *PRAMEF13* | *SNORA105B* | *SNORD131* | *TP53TG3HP* |
| *CFAP100* | *LEXM* | *LINC01619* | *LOC105376599* | *NCBP3* | *PRAMEF18* | *SNORA105C* | *SNORD132* | *TRMO* |
| *CFAP157* | *LINC00374* | *LINC01620* | *LOC105378367* | *NTM-AS1* | *PRAMEF27* | *SNORA107* | *SNORD133* | *USF3* |
| *CFAP161* | *LINC00390* | *LINC01621* | *LOC105378683* | *NUP58* | *PRAMEF33P* | *SNORA108* | *SNORD134* | *WAPL* |
| *CFAP206* | *LINC00430* | *LINS1* | *LOC106660606* | *OLFM5P* | *PRAMEF34P* | *SNORA110* | *SNORD135* | *WDR97* |
| *CFAP73* | *LINC00680* | *LOC100505609* | *LOC106660610* | *OXCT2P1* | *PRAMEF36P* | *SNORA111* | *SNORD136* |  |
| *CFAP77* | *LINC00680-GUSBP4* | *LOC100505771* | *LOC145694* | *PCSK6-AS1* | *PRELID3A* | *SNORA86* | *SNORD137* |  |
| *CRAMP1* | *LINC01022* | *LOC100996624* | *LOC285628* | *PEX5L-AS2* | *PRELID3B* | *SNORA87* | *SNORD140* |  |
| *DPY19L1P2* | *LINC01037* | *LOC101927000* | *LOC728290* | *PLA2G4C-AS1* | *PUM3* | *SNORA88* | *SNORD141A* |  |

Supplementary table 6. Significantly amplified and deleted regional CNAs* in *BRAF*-mt**^#^** and *NRAS*-mt CoM.

| **CNA** | ***BRAF*-mt (n=14)** | ***NRAS*-mt (n=5)** |  | **p value difference between the 2 groups** | **Significantly amplified oncogenes**  **(Fisher’s Exact p value)** | **Significantly deleted tumor suppressor genes**  **(Fisher’s Exact p value)** | **Allelic imbalance** |
| --- | --- | --- | --- | --- | --- | --- | --- |
|  | **No. (%)** | **No. (%)** |  | **(Fisher's Exact p value)** |  |  |  |
| **Significant regional amplifications in *NRAS*-mt CoM** | | | | | | | |
| 17q21.31 | 0 | 3 (60) |  | 0.01 | Nil |  | Hemizygous amplification |
|  | | | | |  |  |  |
| **Significant regional deletions in *BRAF*-mt CoM** | | | | | | | |
| 10q11.21-23.31 | 6-11 (43-78) | 0-1(0-20) |  | 0.6-*0.004* |  | *RASSF4, C10orf99, PTEN (p≤0.04*) | Hemizygous deletion |
| 10q23.2-24.2 | 7-9 (50-64) | 0 |  | - 1. *0.03* |  | *-* |  |
| 10q26.11-26.3 | 7-9 (50-64) | 0 |  | - 1. *0.03* |  | *DMBT1, C10orf90*  (*p≤0.04*) |  |

•: copy number alteration; ^#^: mutant

Supplementary table 7. Summary of immunohistochemistry results of Work Package 2 of CoM

| **Protein & gene location** | **Normal tissue Localisation** | **Total no. of samples examined** | **Deleted copy number CoM** | | | | | | | **Diploid copy number CoM** | | | | | | |
| --- | --- | --- | --- | --- | --- | --- | --- | --- | --- | --- | --- | --- | --- | --- | --- | --- |
|  | | | **No. of samples** | **Total AxB# score** | | | **% Nuclei stained** | | | **No. of samples** | **Total AxB score** | | | **% Nuclei stained** | | |
|  |  |  |  | **Range** | **Mean** | **Median** | **Range** | **Mean** | **Median** |  | **Range** | **Mean** | **Median** | **Range** | **Mean** | **Median** |
| **NEURL1** 10q24.32-25.1 | Cytoplasm & PMº | 15 | 5 | 6-12 | 8.4 | 8 | 0-25 | 11 | 5 | 10 | 4-12 | 8 | 8 | 0-90 | 34.5 | 5 |
| **SUFU** 10q24.32-25.1 | Nucleus | 14 | 5 |  |  |  | 0-100 | 70 | 80 | 9 |  |  |  | 10-100 | 72.2 | 80 |
| **C10orf90** 10q26.13 | Cytoplasm | 13 | 5 | 4-12 | 8 | 8 |  |  |  | 8 | 3-12 | 7.4 | 8 |  |  |  |
| **PDCD4** 10q25.1-25.3 | Cytoplasm & nucleus | 14 | 5 | 0-4 | 1.8 | 1 | 5-95 | 47 | 30 | 9 | 0-12 | 2.7 | 0 | 0-80 | 37.8 | 50 |

#: A= % tumour cells stained, B= intensity if staining; º: Plasma membrane
